# By chance or by choice? Biased attribution of others’ outcomes when social preferences matter

**DOI:** 10.1007/s10683-021-09731-w

**Published:** 2021-09-28

**Authors:** Nisvan Erkal, Lata Gangadharan, Boon Han Koh

**Affiliations:** 1 Department of Economics, University of Melbourne, Melbourne, VIC 3010 Australia; 2 Department of Economics, Monash University, Clayton, VIC 3800 Australia; 3 School of Economics, University of East Anglia, Norwich, NR4 7TJ UK

**Keywords:** Decision-making under risk, Beliefs about others’ decisions, Attribution biases, Social preferences, Consensus effect, Experiments, C92, D91, D81

## Abstract

Decision makers in positions of power often make unobserved choices under risk and uncertainty. In many cases, they face a trade-off between maximizing their own payoff and those of other individuals. What inferences are made in such instances about their choices when only outcomes are observable? We conduct two experiments that investigate whether outcomes are attributed to luck or choices. Decision makers choose between two investment options, where the more costly option has a higher chance of delivering a good outcome (that is, a higher payoff) for the group. We show that attribution biases exist in the evaluation of good outcomes. On average, good outcomes of decision makers are attributed more to luck as compared to bad outcomes. This asymmetry implies that decision makers get too little credit for their successes. The biases are exhibited by those individuals who make or would make the less prosocial choice for the group as decision makers, suggesting that a consensus effect may be shaping both the belief formation and updating processes.

**Supplementary Information:**

The online version contains supplementary material available at 10.1007/s10683-021-09731-w.

## Introduction

In many environments, the determinants of outcomes are not observable. Decision makers make unobserved choices under risk and uncertainty, and outcomes are determined by a combination of their choices and luck. For instance, a firm’s profits are determined by both the business strategies taken by its managers and the macroeconomic factors that are beyond their control. How are outcomes evaluated in such situations? Are there systematic biases in the attribution of outcomes to the decision makers’ choices versus luck? Do they receive too little or too much credit?

We explore these questions in a leadership context where the choices decision makers make under risk affect their own payoffs as well as those of other individuals. Leadership is often about decision making for others and inherently involves assuming responsibility for the outcomes of others (Ertac & Gurdal, 2012; Edelson et al., 2018). In many cases, decision makers face a trade-off between maximizing their own payoff and those of other individuals. For example, society’s growing demand for corporate social responsibility, defined as sacrificing firm profits for social interest, demonstrates how decision makers in positions of power are expected to engage in prosocial activity (Bénabou & Tirole, 2010).

We report findings from two experiments designed to examine how individuals who are affected by the choices of the decision makers form inferences about the decision makers. Our experimental design emphasizes the role social preferences play in leadership and aims to analyze the inferences formed about this important personality trait of decision makers. Uncovering biases in the attribution of outcomes is important for understanding the attitudes towards decision makers and the decision-making environment.^1^

In Experiment 1, individuals in their role as decision makers make an investment choice on behalf of their group. They choose between two investment options with binary outcomes. The outcome to the group depends on both the decision maker’s choice, which is unobservable to the other group members, and luck. A high investment leads to a higher probability of the good outcome for the group but comes at a higher private cost to the decision maker. Hence, one can also think of the high investment decision as a costly effort choice made by the decision maker that increases the group’s surplus at a personal cost. Consequently, decision makers’ choices are affected by their social preferences. Using this design, we examine the group members’ initial beliefs about the decision maker’s type, and how these beliefs are updated after observing the outcome of the choice made by the decision maker.

In our analysis of belief updating, we examine two issues. First, we study biases in the way prior beliefs are treated in the updating process when group members form inferences about decision makers’ prosocial preferences. More precisely, taking Bayes’ rule as the benchmark, we ask whether group members suffer from base-rate neglect (i.e., put too little weight on their prior beliefs) or confirmatory bias (i.e., put too much weight on their prior beliefs) relative to a Bayesian.^2^ Second, we examine whether group members respond too little or too much to new information about the choice made by the decision maker, and whether there is an asymmetry in the way good and bad outcomes are treated. Responding too little to a good (bad) outcome, for example, would imply that they believe decision makers act selfishly (prosocially) and luck plays a bigger role in determining outcomes. Hence, if members exhibit a bigger response to bad outcomes (as compared to good outcomes), then this implies that they are more likely to blame the decision maker for acting selfishly when they see a bad outcome, but they do not attribute a good outcome to the decision maker’s prosociality.

In Experiment 1, we find that group members consistently suffer from base-rate neglect. This indicates, for example, that members who are initially more optimistic about the likelihood that the decision maker made a high investment decision tend to over-update their beliefs about the decision maker’s behavior when they observe a bad outcome. After accounting for base-rate neglect, we find that on average, members under-respond to good outcomes and attribute them more to luck as compared to a Bayesian. In contrast, their response to bad outcomes is similar to a Bayesian. This asymmetry implies that members on average attribute good outcomes more to luck and bad outcomes more to the decision maker’s selfish choice. As a result, decision makers get too little credit for their successes.

We also consider whether members’ belief-updating behavior depends on the process by which the decision maker is selected. For instance, it may be the case that members are more likely to blame decision makers for bad outcomes if they are not appointed by the group. Consistent with our theoretical framework, we find that the appointment mechanism affects the initial beliefs formed about the decision maker’s type. For example, members believe that a group-appointed decision maker is more likely to act in the group’s interest as compared to a randomly appointed decision maker. However, once we control for the impact of the appointment mechanism on the initial beliefs, we find that the appointment mechanism has no additional impact on the updated beliefs.

A feature of our design in Experiment 1 is that the decision makers’ choices and members’ beliefs are elicited using the strategy method, where all individuals first make choices as decision makers before reporting their beliefs as group members. This allows us to examine the relationship between individuals’ choices as decision makers and their attribution of the decision makers’ outcomes as members. Using this design, we uncover that the asymmetry we identify in the evaluation of good and bad outcomes is driven by those individuals who make the less prosocial choice for the group. That is, those who make lower investment choices as decision makers are more likely to attribute others’ good outcomes to luck. This suggests that a consensus effect may be at play as individuals use their own behavior as the basis for updating their beliefs about others (Ross et al., 1977; Marks & Miller, 1987; Dawes, 1989).^3^

We explore this result further in Experiment 2, where participants no longer play both roles in the experiment. We are interested in investigating whether the biases we observe in Experiment 1 still exist when group members do not have experience making choices as decision makers.^4^ Participants are informed at the beginning of the experiment whether they have been assigned as a decision maker or a group member. These roles do not change throughout the experiment.

To investigate whether different types (i.e., prosocial versus selfish individuals) form and update their beliefs differently, we ask group members to report their beliefs *before* asking them to indicate, hypothetically, what their investment decision would have been if they were the decision maker. This allows us to test whether there exists a correlation between each group member’s type and their belief irrespective of whether they play both roles or just one. That is, by eliciting these hypothetical decisions after the belief-elicitation stage, we are still able to examine the relationship between individuals’ effort choices as decision makers and their beliefs as members.

Interestingly, a correlation between the attribution of good outcomes to luck and what members would have chosen as decision makers also emerges in Experiment 2. That is, those members who are more likely to attribute good outcomes to luck are also more likely to state afterwards that they would have chosen low effort if they were placed in the position of the decision maker. This leads us to conclude that with and without the experience of acting as a decision maker, the same biases exist and seem to be driven by a consensus effect.

Our paper is related to three strands of the literature. First, our study substantially advances the research on attribution biases in beliefs in both economics and psychology. Studies in experimental economics have analyzed biases in beliefs and information processing by focusing mainly on ego-related beliefs, i.e., beliefs about one’s own ability or physical attributes where one’s ego can play a big role in shaping their beliefs (Eil & Rao, 2011; Ertac, 2011; Grossman & Owens, 2012; Möbius et al., 2014; Coutts, 2019). Both Eil and Rao (2011) and Möbius et al. (2014) find evidence of asymmetric updating, where agents are more responsive to good news than to bad news about their own performance in an IQ test or a beauty task. While Grossman and Owens (2012) find no evidence of asymmetry, Ertac (2011) and Coutts (2019) find that individuals tend to overweigh bad news.^5^

The related literature in psychology has mainly focused on self-serving biases in the attribution of own versus others’ outcomes (see, e.g., Miller and Ross, 1975). Consistent with our findings, individuals tend to attribute others’ good outcomes to exogenous factors (such as luck). In comparison, they are more likely to attribute their own good outcomes to endogenous factors (such as ability). Similarly, Pettigrew (1979) finds that good outcomes of out-group members are attributed to luck, but the opposite pattern emerges for in-group members.

Our novelty in relation to this strand of the literature in both economics and psychology is that we focus on the evaluation of others’ outcomes in a context where decision making is shaped by social preferences. We show that good and bad outcomes are treated asymmetrically in this case also, and attribution biases exist in the case of good outcomes only. Moreover, our findings reveal that individuals’ evaluation of others’ prosociality tend to be correlated with their own behavior.

Second, our work is related to the literature on outcome biases, where researchers also find asymmetric evaluation of others’ good and bad outcomes. However, it is assumed in this literature that all determinants of outcomes are fully observable. Despite this, good outcomes are treated more favorably than bad outcomes, suggesting that evaluators are biased by luck (see, e.g., Charness and Levine, 2007; Gurdal et al., 2013; Brownback and Kuhn, 2019). Our research extends this literature by considering the (arguably more common) setup where determinants of outcomes are not observable.

Finally, our paper is related to the literature which investigates how individuals respond to others’ favorable and unfavorable outcomes under uncertainty in contexts such as CEO compensation (Bertrand and Mullainathan, 2001; Leone et al., 2006), political elections (Wolfers, 2007; Cole et al., 2012), medical referrals (Sarsons, 2019), and soccer (Gauriot & Page, 2019).^6^ In addition, a significant amount of attention has been devoted to preferences for redistribution under uncertainty. While some papers investigate the link between redistribution and beliefs about the determinants of inequality (Fong, 2001; Linos & West, 2003; Aarøe & Petersen, 2014; Alesina et al., 2018; Rey-Biel et al., 2018), others aim to identify the causal effect of varying the source of inequality (such as merit versus luck) on the level of redistribution (Cappelen et al., 2007; Almås et al., 2010; Durante et al., 2014; Almås et al., 2020). A key objective of all these studies is to examine how performance is evaluated under uncertainty. In contrast, our aim is to focus on the belief formation and updating process, and to study specifically the biases which may characterize it. Our experimental setting gives us the opportunity to examine attribution biases in a controlled environment with an objective signal generating process.

## Experiment 1

### Experimental design

**Fig. 1:**
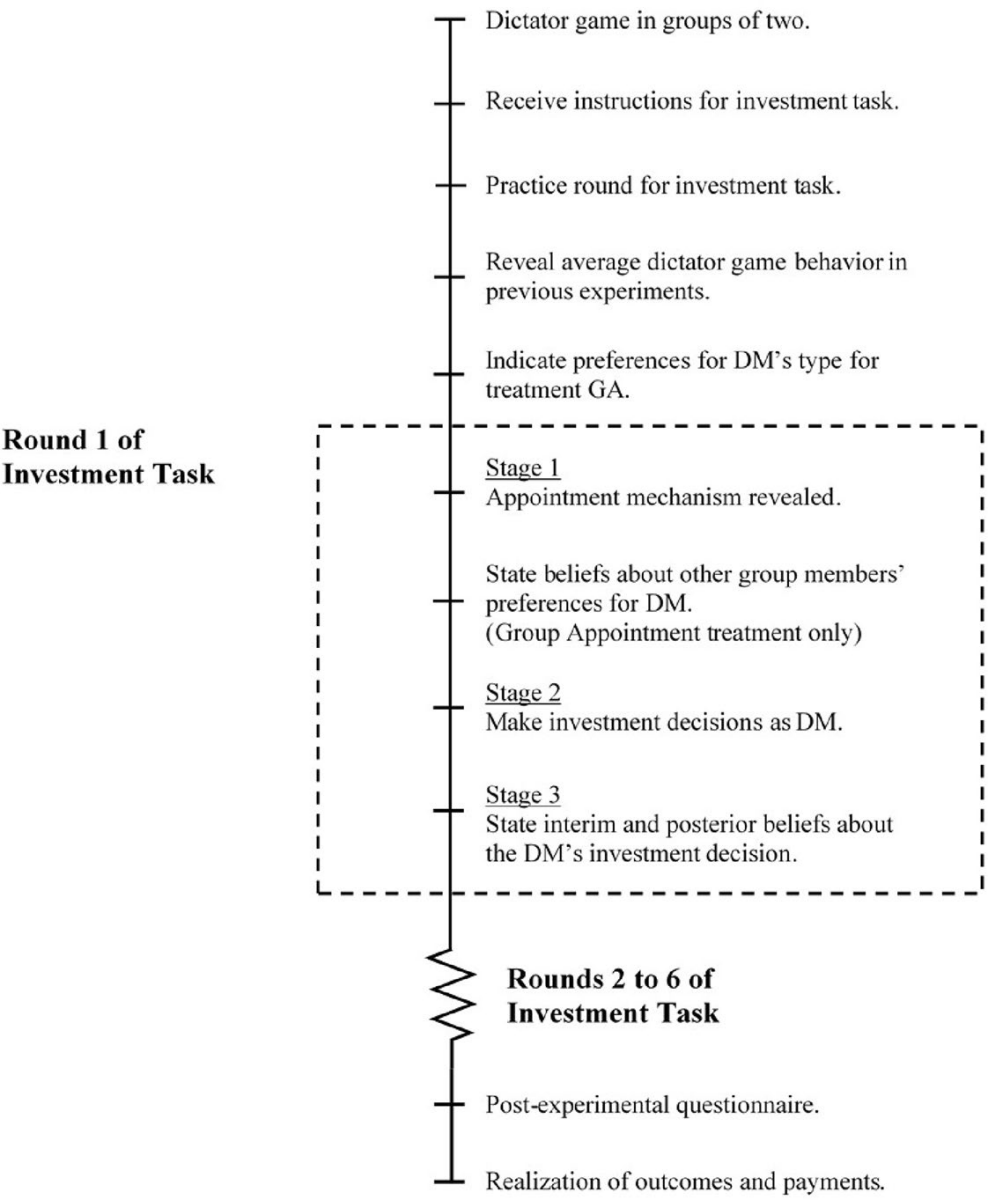
Overview of Experiment 1

Figure 1 presents an overview of the experiment. The main task in the experiment is the investment task, which we explain in Sect. 2.1.1. According to our theoretical framework, decisions in the investment task are shaped by the subjects’ social preferences. Hence, to elicit their social preferences, subjects also play the dictator game in groups of two. Each subject is given 300 Experimental Currency Units (ECU) and asked to allocate this endowment between themselves and their matched partner. Both subjects within the pair make allocation decisions as the dictator. They are told that one of the decisions will be randomly chosen at the end of the experiment to determine the final allocation of the given endowment within each pair. Once subjects play the dictator game, they receive instructions for the investment task.^7^

#### Investment task

The experiment features a within-subject treatment design, where subjects play six repeated rounds of the investment task. In each round, subjects are re-matched to a new group with two other individuals (perfect stranger matching). Within each group, there is a decision maker (referred to as the DM in the rest of the paper) who makes an unobservable investment decision on behalf of the group. In the experiment, we label the DM as the leader.

Decisions are elicited using a strategy method. In each round, all subjects make their investment decisions assuming that they have been assigned to be the DM, and then state their beliefs about their DM’s investment decision assuming that someone else in the group has been assigned to be the DM. This allows us to analyze whether beliefs are correlated with individuals’ own decisions.^8^ No feedback is given during the entire experiment. Subjects are informed whether they were assigned the role of the DM at the end of the experiment.

As shown in Fig. 1, each round of the investment task consists of three stages, which we now explain in detail.

*Stage 1: Appointment of DM*. Group members’ unconditional beliefs about their DM’s investment decision can potentially depend on how the DM is appointed. We consider four mechanisms of appointing the DM. The appointment mechanism changes across the rounds, varying the initial beliefs members hold. This allows us to examine whether members’ updating behavior depends on the distribution of their initial beliefs. For example, it may be the case that members are more likely to blame DMs for their failures if the DM is not appointed by the group.

At the beginning of each round, subjects are informed which mechanism will be employed in that round. In three of the appointment mechanisms, the DM is appointed exogenously. In the random assignment mechanism (treatment RA), each individual has an equal chance of being appointed as the DM. In the low and high assignment mechanisms (treatments LA and HA), subjects are informed that the group member who allocated the least and the highest amount to their matched partner in the dictator game, respectively, would be appointed as the DM.^9^ Ties are broken randomly.

The fourth mechanism is the group appointment mechanism (treatment GA). Before beginning the first round of the investment task, each group member is asked to indicate whether they prefer: (i) to appoint the member who allocated the lowest amount to their matched partner in the dictator game; (ii) to appoint the member who allocated the highest amount to their matched partner in the dictator game; or (iii) to randomly select one member to be the DM. In addition, the subjects are asked to state their beliefs about the other two group members’ preferences on which appointment mechanism to use.^10^ To appoint the DM, the computer randomly picks one group member. This member’s decision is used to determine which of the *other* two members will be the DM. This ensures that there is no scope for strategic behavior in that subjects are unable to influence their probability of being the DM through their decisions.^11^ This is especially important in our set-up because, as explained later, there is a clear advantage to being the DM.

*Stage 2: DM’s investment decision.* In the second stage of the investment task, each subject is asked to make an investment decision on behalf of the group.

**Fig. 2:**
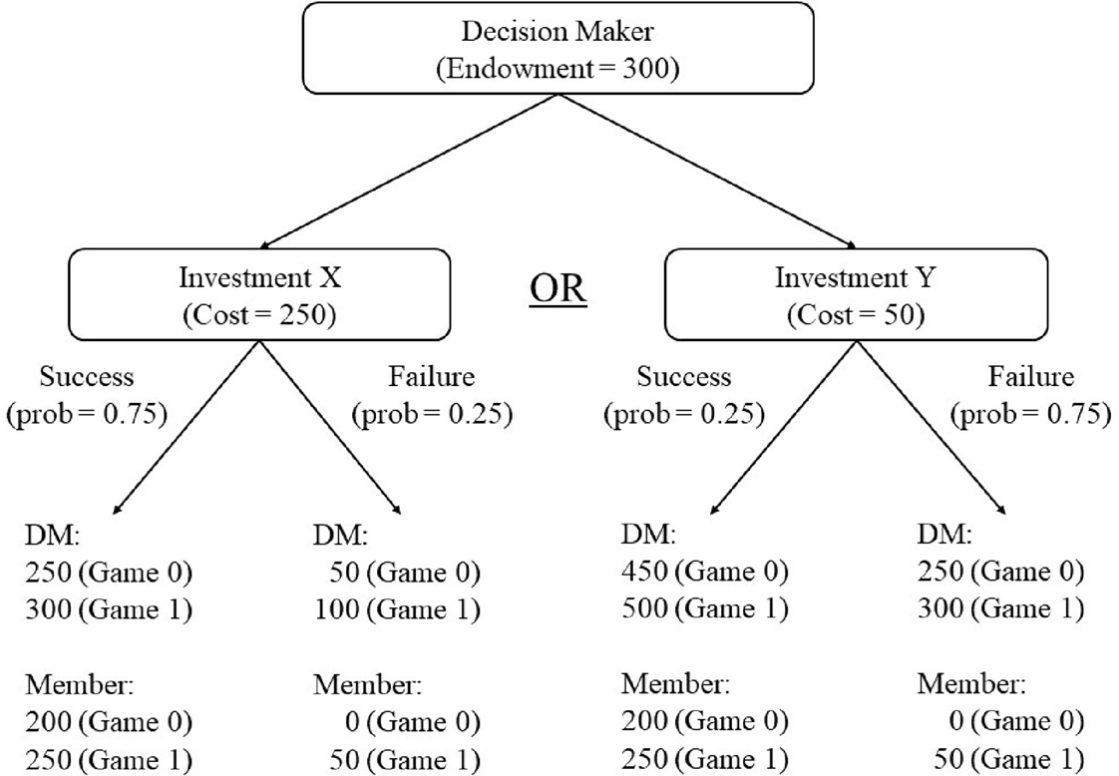
Investment task

The DM is given an individual endowment of 300 ECU and chooses between two investment options that will affect the payoffs of all the group members. The two investment options, given in Fig. 2, are: (i) Investment X, which corresponds to a high effort level; and (ii) Investment Y, which corresponds to a low effort level. Both investment options yield the same high return if they succeed and the same low return if they fail. However, they differ in their probability of success/failure, and in their cost to the DM. Investment X succeeds with a probability of 0.75 and costs the DM 250 ECU, while Investment Y succeeds with a probability of 0.25 and costs the DM 50 ECU. Subjects are informed that the DM’s investment decision will not be revealed to the group members. They only learn the outcome of the investment in the round randomly chosen for payment at the end of the experiment.

The returns from the two investment options are assumed to take the following values. In Game 1, the investment provides a return of 750 ECU for the group if it succeeds and 150 ECU if it fails. Note that the subjects’ investment decisions as the DM and their beliefs as members about the DM’s investment decision may be sensitive to the returns associated with the investment options. For instance, some subjects may be averse to choosing Investment Y if the members will receive a payoff of zero in case of failure. For this reason, we also consider Game 0, where the investment provides a return of 600 ECU if it succeeds and 0 ECU if it fails.

The return from the investment is distributed evenly between the DM and the two group members. The amount determines each group member’s final payoff, except for the DM’s. The DM’s final payoff is equal to the sum of the endowment and the share of the return from the investment minus the cost of investment. These final payoffs are given at the bottom of Fig. 2.

*Stage 3: Elicitation of beliefs of group members*. In the third stage of the investment task, subjects are asked to assume the role of group members and state their beliefs on the likelihood that the DM (i.e., one of the other two players in their group) has chosen Investment X. We elicit beliefs in the form of frequencies rather than probabilities.^12^ When stating their beliefs, subjects are required to enter an integer number between 0 and 100.

We elicit two sets of beliefs from each subject.^13^ First, each subject is asked to state their unconditional belief that the DM has chosen Investment X. Given that the subjects form these beliefs after being informed of the appointment mechanism, we refer to these unconditional beliefs as the members’ *interim* beliefs. Second, each subject is asked to state their beliefs conditional on observing whether the investment chosen by the DM has succeeded or failed. We refer to these beliefs as the members’ *posterior* beliefs. We do not impose any restrictions on their posterior beliefs. The group members can state any belief they want, regardless of what their interim beliefs are.

Subjects are paid for either their interim belief or their posterior beliefs. Beliefs are incentivized using the binarized scoring rule (BSR). We use the BSR because it incentives truth-telling independent of the subjects’ risk preferences (Hossain & Okui, 2013; Erkal et al., 2020). It is a modified version of the quadratic scoring rule with a binary lottery procedure, where the distance between a subject’s belief report and the DM’s investment decision determines the probability of receiving a fixed amount (10 ECU in this case). As the subject’s reported belief gets further away from the DM’s investment decision, the probability of receiving the fixed payment gets lower.^14^

#### Procedures and payment

The experiments were conducted in the Experimental Economics Laboratory at the University of Melbourne (*E*^2^*MU*) and programmed using z-Tree (Fischbacher, 2007). We ran 10 sessions with 24 to 30 subjects in each session. A total of 282 participants, mostly students from the University of Melbourne, were recruited using ORSEE (Greiner, 2015).^15^ Each session lasted between 90 and 120 min.

To ensure that the subjects fully understood the tasks, the experimenter verbally summarized the instructions after the subjects finished reading the printed instructions. Subjects completed a set of control questions and participated in a practice round using treatment GA and Game 0 before beginning the actual investment task. For Game 0, we implemented treatments LA and HA only since, as explained in the next section, theory suggests that the difference in interim beliefs should be the greatest between these two treatments. We implemented all four appointment mechanisms for Game 1, which allows us to study the subjects’ behavior across different mechanisms using the same set of parameters.

The order between treatments was changed to control for potential order effects. However, since our main focus is the treatments associated with Game 1, Game 0 was always implemented in Rounds 1 and 2 while Game 1 was always implemented in Rounds 3 to 6. Table 1 summarizes the order of the treatments in each session. In each cell of the table, the first two letters denote the appointment mechanism, while the Arabic numeral at the end denotes the game faced by the subjects in the corresponding round within the session.^16^

**Table 1:** Order of treatments for each experiment session

Session	# Subjects	Round #						
		Practice	1	2	3	4	5	6
1, 5	60	GA0	LA0	HA0	RA1	LA1	HA1	GA1
2, 6	60	GA0	HA0	LA0	RA1	HA1	LA1	GA1
3, 8	54	GA0	LA0	HA0	GA1	LA1	HA1	RA1
4, 7	54	GA0	HA0	LA0	GA1	HA1	LA1	RA1
9, 10	54	GA0	LA0	HA0	LA1	HA1	GA1	RA1

At the end of the experiment, subjects were invited to complete a brief questionnaire which included demographic questions, questions about their decisions during the experiment, and an incentivized one-shot risk task (Gneezy & Potters 1997) to elicit their risk preferences. Subjects were paid for either the dictator game or the investment task. If they were paid for the investment task, then we paid them for their decisions in one of the six rounds. For the chosen round, a DM was appointed according to the corresponding treatment and the DM was paid only for their investment decision. The other two members were paid for their DM’s decision as well as their stated beliefs. Earnings were converted to cash at the conclusion of the session at the rate 10 ECU = 1 AUD. Overall, subjects earned between $10 and $76, with the mean earnings being $ 34.07. Subjects’ earnings also included a show-up fee of $10.

### Theoretical framework

In this section, we provide a simple theoretical framework to evaluate how beliefs will be formed under the different appointment mechanisms.

#### DM’s effort choice

Players maximize expected utility and are differentiated based on their other-regarding preferences. Let $\beta _i\in [0,1]$ denote the type of player *i*. It is a private draw from a distribution $F(\beta )$ with density $f(\beta )$. $F(\beta )$ is common knowledge.^17^

Players are randomly assigned to groups of size $N>2$. The DM in each group makes an effort choice $e\in \{e_L,e_H\}$ at cost $c\in \{c_L,c_H\}$ which is deducted from an initial endowment $\omega$ that the DM receives. Assume that $\omega \ge c_H>c_L>0$. There are two possible team outputs, $Q\in \{Q_L,Q_H\}$, where $Q_H>Q_L$, and the DM’s effort choice determines the probability with which each output level will be realized. A high effort choice leads to the high output level with a higher probability, but it costs more to the DM. Specifically, a high effort choice $e_H$ leads to an output $Q_H$ with probability *p*, where $p\in (0.5,1)$, while a low effort choice $e_L$ leads to an output $Q_H$ with probability $1-p$.

For a given outcome *Q*, each member in the group receives $\frac{Q}{N}$ and the utility of the DM is given by

1 $$\begin{aligned} U=u\left( \frac{Q}{N}+\omega -c\right) +\beta \cdot \sum _j v_j\left( \frac{\rm Q}{N}\right), \end{aligned}$$

where $u(\cdot )$ is the direct utility the DM receives from their own monetary payoff and $v_j(\cdot )$ represents the utility member *j* receives from their own monetary payoff. We assume $u'(\cdot )>0$, $v_j'(\cdot )>0$, and $\beta$ denotes the weight the DM puts on the utilities of the other group members.^18^

DMs maximize their expected utility and choose $e_H$ over $e_L$ if $EU(e_H)\ge EU(e_L)$. In the experimental design, we refer to $e_H$ and $e_L$ as Investment X and Investment Y, respectively. The choice of parameters in Game 0 and Game 1 are $N=3$, $\omega =300$, $p=0.75$, $Q_H=$ 750 (Game 1) or 600 (Game 0), $Q_L=$ 150 (Game 1) or 0 (Game 0), $c_H=250$, and $c_L=50$. Given these parameter choices, if $\beta =0$, then the DMs only care about their own payoff and choose $e_L$ since $EU(e_H)-EU(e_L) = p\big [u\big (\frac{Q_H}{N}+\omega -c_H\big ) - u\big (\frac{Q_L}{N}+\omega -c_L\big )\big ] + (1-p)\big [u\big (\frac{Q_L}{N}+\omega -c_H\big ) - u\big (\frac{Q_H}{N}+\omega -c_L\big )\big ] < 0$.^19^ For $\beta >0$, $EU(e_H)\ge EU(e_L)$ holds if

$$\begin{aligned} \beta \ge \beta ^* \equiv \dfrac{ \left[ \begin{array}{ll}{p\left[ u\left( \frac{Q_H}{N}+\omega -c_H\right) -u\left( \frac{Q_L}{N}+\omega -c_L\right) \right] }\\ \quad {+(1-p)\left[ u\left( \frac{Q_L}{N}+\omega -c_H\right) -u\left( \frac{Q_H}{N}+\omega -c_L\right) \right] }\end{array} \right] }{ (1-2p) \sum _j \left[ v_j\left( \frac{Q_H}{N}\right) - v_j\left( \frac{Q_L}{N}\right) \right] } . \end{aligned}$$

Intuitively, DMs choose high effort if they care sufficiently about the payoffs of the other group members.^20^ In the experiment, subjects’ decisions in the dictator game provide a proxy for their types ($\beta _i$). We use the dictator game since it is widely used in the literature to measure social preferences.

#### Information and beliefs

*Members’ interim beliefs.* We first consider the members’ interim beliefs about their DM’s type after observing the appointment mechanism. Specifically, we are interested in each member’s belief that the DM is of type $\beta \ge \beta ^*$, which corresponds to the likelihood that the DM chooses $e_H$ over $e_L$. We denote member *i*’s interim belief after observing appointment mechanism $\Psi \in \{RA,LA,HA,GA\}$ as $\mu _i^{\Psi }$.

Our first testable prediction is about the ranking of the members’ interim beliefs under the different appointment mechanisms:

##### Hypothesis 1

$$\mu _i^{LA}\le \mu _i^{RA}\le \mu _i^{GA}\le \mu _i^{HA}$$.

The proof is in Appendix C. In treatment GA, all players prefer to have the highest type appointed as the DM. This is because all group members want the DM to choose $e_{H}$ which maximizes their expected payoffs. Although this implies that the beliefs under treatments GA and HA should be the same, the difference stated in the hypothesis is due to the implementation strategy we follow in treatment GA. Specifically, the highest type in the group will not necessarily be appointed as the DM under treatment GA if his/her appointment decision is randomly picked to be implemented. Hence, $\mu _i^{GA}\le \mu _i^{HA}$.

*Members’ posterior beliefs.* We next consider how members update their beliefs about their DM’s type after observing the outcome. The outcome $Q\in \{Q_L,Q_H\}$ is a signal that members receive about the DM’s type. Note that $Pr(Q_L|\beta <\beta ^*)=Pr(Q_H|\beta \ge \beta ^*)=p$ and $Pr(Q_L|\beta \ge \beta ^*)=Pr(Q_{H}|\beta <\beta ^*)=1-p$.

We denote group member *i*’s unbiased posterior belief of the DM’s type, given a signal *Q*, as $\phi _{i}^{\Psi }|_{Q}$. Specifically, suppose the members receive a signal $Q=Q_H$. Using Bayes’ rule, member *i*’s posterior belief is given by

$$\begin{aligned} \phi _i^{\Psi }|_{Q_H}=\frac{\mu _i^{\Psi } \cdot Pr(Q_H|\beta \ge \beta ^*)}{Pr(Q_H)}=\frac{\mu _i^{\Psi }p}{\mu _i^{\Psi }p+(1-\mu _i^{\Psi })(1-p)}. \end{aligned}$$

$\phi _i^{\Psi }|_{Q_L}$ is defined in a similar way.

We test the null hypothesis that the members will be unbiased (i.e., Bayesian) when they update their beliefs:

##### Hypothesis 2

Group members behave like Bayesian agents when updating their beliefs about the DM under all of the appointment mechanisms.

We explain the econometric framework that we use to test Hypothesis 2 empirically in Sect. 2.3.3. If we detect deviations from the Bayesian benchmark (e.g., Tversky & Kahneman, 1974), our design allows us to investigate these deviations further in two ways. First, we can test whether the deviations vary across the different appointment mechanisms. That is, we can observe, for example, whether being appointed by the group (treatment GA) has an impact on the way members update their beliefs about the DM. Second, since we employ a strategy method, we can examine whether there is a correlation between subjects’ decisions as DMs and their beliefs (initial and updated) about the DM. Such a correlation can be explained by a consensus effect. For example, a subject who chooses $e_H$ may be more likely to believe that their DM has also chosen $e_H$.

### Results

Since there were no interactions between the group members during the experiment and no feedback was given to the subjects from the previous rounds, our unit of observation is at the subject level. For the main analyses in this paper, we pool data from the Game 0 and Game 1 treatments.^21^ For robustness, we show in Appendix D.1 that the main conclusions do not change when we consider the Game 1 treatments only.

#### The dictator game as a proxy for an individual’s type

We conjecture in Sect. 2.2 that subjects’ behavior in the dictator game is a proxy for their type. That is, subjects who transfer more of their endowment to their matched partner in the dictator game are more likely to choose $e_H$ when they are in the role of the DM. As our testable hypotheses depend on this relationship between the DM’s type and their effort choice, we first examine if it holds.

**Fig. 3:**
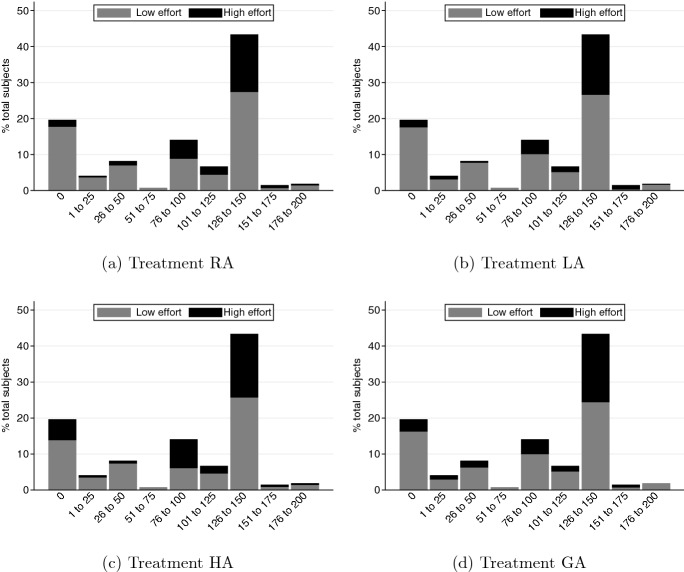
DM’s effort choice in Game 1 against dictator game behavior

Figure 3 presents the distribution of subjects’ decisions in the dictator game against their effort choices across different appointment mechanisms. Because the subjects only participate in the dictator game once, the distribution of transfers are the same across the different treatments. Within each panel in Fig. 3, the black bars represent the proportion of DMs who choose high effort ($e_H$) while the gray bars represent the DMs who choose low effort ($e_L$).

A clear pattern that emerges is that DMs who are more prosocial in the dictator game are also the ones who are more likely to choose the investment option that is in the interest of the group (i.e., high effort). This pattern is consistent across the different appointment mechanisms.^22^ The correlation between the DM’s behavior in the dictator game and their effort choice are statistically significantly positive in all treatments. The Spearman’s rank correlation coefficients and corresponding *p* values are: (i) RA: 0.233, *p* value < 0.001; (ii) LA: 0.262, *p* value < 0.001; (iii) HA: 0.096, *p* value = 0.025; and (iv) GA: 0.183, *p* value = 0.003.

Table 2 presents marginal-effects estimates from a probit model for the relationship between the subjects’ decisions as DMs in the investment task and their dictator game behavior. In the regression analysis, we control for order effects, the subjects’ behavior in the risk task, the appointment mechanisms, and Game 1. We find a statistically significant and positive relationship between the DM’s decision in the dictator game and their decision to choose high effort in the investment task (*p* value < 0.001). A DM who transfers 1% more of their endowment to their matched partner in the dictator game is 0.4% more likely to choose $e_H$ in the investment task on average. In addition, consistent with our expectations about the DM’s behavior between the Game 0 and Game 1 treatments, we observe that subjects are 6.7% less likely to choose $e_H$ in Game 1 on average, and this effect is statistically significant (*p* value = 0.003).^23^

**Table 2:** Regression of DM’s effort choice

	Dependent variable: =1 if DM chooses $e_H$
Variables	(1)
% endowment transferred in DG	0.004***
	(0.001)
% endowment invested in RT	−0.001
	(0.001)
Treatment LA	−0.044*
	(0.026)
Treatment HA	0.046
	(0.029)
Treatment GA	0.040
	(0.029)
Game 1	−0.067***
	(0.022)
Order Effects	Y
Observations	1,632
# subjects (clusters)	272

Marginal effects of probit model reported. Robust standard errors in parentheses. Standard errors are clustered at the subject level.

DG: Dictator Game; RT: Risk Task.

*** *p*<0.01, ** *p*<0.05, * *p*<0.10.

**Fig. 4:**
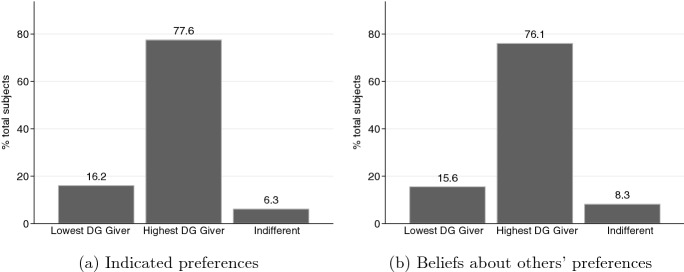
Preferences for DM under Treatment GA

The established link between dictator game behavior and effort choices implies that subjects’ preferences in treatment GA should be for the highest type to be appointed as the DM. Figure 4 presents the subjects’ preferences for their DM’s type under treatment GA (panel a) and their beliefs about the preferences of the other group members (panel b). The majority of the subjects (77.6%) prefer to have the individual who made the highest transfer in the dictator game to be the DM of their group. Moreover, the majority of the subjects (76.1%) believe that the other members of their group prefer to appoint the individual who made the highest transfer as the DM.

#### Analysis of interim beliefs

**Fig. 5:**
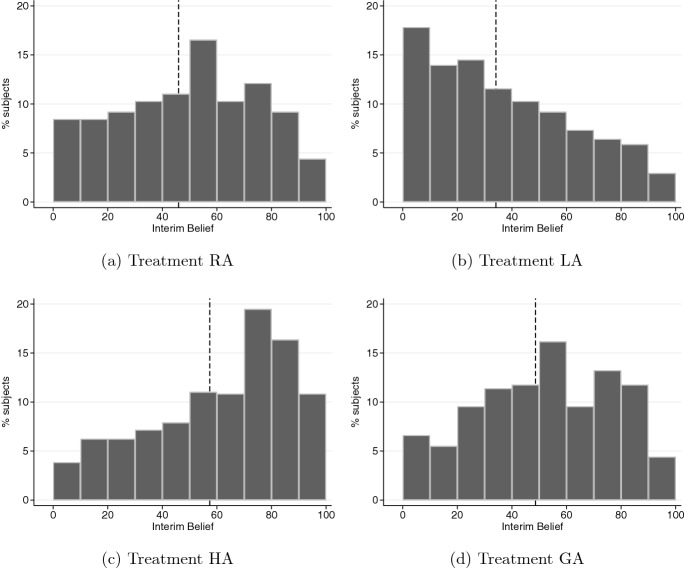
Distributions of group members’ interim beliefs

We next examine the members’ interim beliefs after they observe the appointment mechanism but prior to observing the DM’s outcomes. In all of our analyses, belief is a variable that takes an integer value in [0, 100], where a higher belief implies that the member thinks the DM is more likely to have chosen high effort ($e_H$). Figure 5 presents the distributions of the members’ interim beliefs by treatment. In each panel, the dashed line represents the mean interim belief.

The histograms in Fig 5 suggest that group members respond to the mechanism used to appoint the DM, as stated in Hypothesis 1. In treatment RA, the DM is randomly assigned and the members’ beliefs are approximately centered on 50%, with a mean of 45.94% (panel a). In contrast, the distribution of interim beliefs is highly skewed to the right in treatment LA with a mean of 34.15% (panel b), and to the left in treatment HA with a mean of 57.40% (panel c). When the DM is appointed based on the preferences of the group in treatment GA (panel d), the distribution of interim beliefs shifts slightly to the right relative to that in treatment RA, and the average interim belief increases to 48.65% which is lower than that in treatment HA.^24^

Table 3 presents OLS estimates for the regressions of interim beliefs against treatment variables, controlling for Game 1, order effects (in columns 1 and 3), and individual fixed effects (in columns 2 and 4). In all the specifications, treatment RA is the comparison group. The last row presents the results of a Wald test of equality between treatments GA and HA. The coefficient estimates in columns (1) and (2) support our conclusions from the non-parametric analysis. We also find that the members’ beliefs are on average lower in Game 1 treatments than in Game 0 treatments. This difference is statistically significant in both columns (1) and (2) (*p* value = 0.005 in both columns).

**Table 3:** Regression of members’ interim belief

Variables	Dependent variable: Interim belief			
	(1)	(2)	(3)	(4)
Treatment LA	−13.268***	−13.268***	−12.237***	−12.646***
	(1.417)	(1.416)	(1.403)	(1.372)
Treatment HA	9.982***	9.982***	8.950***	9.359***
	(1.311)	(1.309)	(1.263)	(1.246)
Treatment GA	2.717**	2.717**	1.857	2.198*
	(1.355)	(1.353)	(1.273)	(1.271)
Chooses high effort as DM			23.382***	14.109***
			(1.848)	(1.588)
% endowment invested in RT	−0.104**		−0.073**	
	(0.043)		(0.036)	
Game 1	−2.952***	−2.952***	−1.405	-2.018**
	(1.041)	(1.040)	(0.955)	(0.964)
Constant	59.182***	48.890***	48.117***	44.066***
	(3.916)	(1.237)	(3.534)	(1.243)
Order Effects	Y	N	Y	N
Individual FE	N	Y	N	Y
Observations	1,632	1,632	1,632	1,632
# subjects (clusters)	272	272	272	272
R-squared	0.137	0.251	0.286	0.305
*Test of GA = HA*				
test statistic	5.604	5.610	5.738	5.860
*p* value	${<}$ 0.001	${<}$ 0.001	${<}$ 0.001	${<}$ 0.001

Robust standard errors clustered at the subject level in parentheses. For all regressions, treatment RA is the reference treatment.

RT: Risk Task.

*** *p*<0.01, ** *p*<0.05, * *p*<0.10.

In columns (3) and (4), we control for the subjects’ own decision as a DM. A subject who chooses to exert high effort when placed in the position of the DM under a specific appointment mechanism is also more likely, as a group member, to expect the DM to choose high effort under the same appointment mechanism. This effect is statistically significant (*p* value < 0.001 in both columns).^25^ The treatment effects remain similar in both direction and magnitude after controlling for the consensus effect, although the estimates for treatment GA are now statistically insignificant in column (3) and marginally statistically significant in column (4) (*p* values = 0.146 and 0.085, respectively).

We summarize our results in support for Hypothesis 1 as follows:

##### Result 1

Group members respond to the appointment mechanism in their interim beliefs. The interim beliefs are the lowest in treatment LA and the highest in treatment HA. The interim beliefs in treatment RA are lower than those in treatment GA.

#### Analysis of posterior beliefs

*Estimation strategy for posterior beliefs*. To test Hypothesis 2 and analyze updating behavior, we estimate the following equation:

2 $$\begin{aligned} \mathrm {logit}(\hat{\phi _i^{\Psi }}|_{Q}) = \delta \, \mathrm {logit}(\hat{\mu _i^{\Psi }}) + \gamma _G\, I(Q=Q_H) \cdot \mathrm {logit}(p) + \gamma _B\, I(Q=Q_L) \cdot \mathrm {logit}(1-p) + \varepsilon _i , \end{aligned}$$

where $\mathrm {logit}(x)\equiv \log (x)/\log (1-x)$ and $\varepsilon _i$ captures non-systematic errors. This specification allows us to determine the weights members place on their interim beliefs and the signals they receive.^26^ Note that $\delta =\gamma _G=\gamma _B=1$ corresponds to the Bayesian benchmark. Hypothesis 2 states that $\delta =\gamma _G=\gamma _B=1$ for each appointment mechanism.

Any deviation in the parameters from 1 is interpreted as non-Bayesian updating behavior. Appendix E provides detailed explanations of the interpretations of these parameters. First, $\delta$ captures the weight that group members place on their interim belief in the updating process. If $\delta <1$ ($\delta >1$), then members suffer from *base-rate neglect* (*confirmatory bias*) in that they place too little (too much) weight on their interim belief.

Next, $\gamma _G$ and $\gamma _B$ capture the extent to which members respond to signals of good outcome and bad outcome from the DM, respectively. $\gamma _G>1$ or $\gamma _B>1$ implies that members are, on average, over-responsive to a good or a bad signal, respectively, relative to a Bayesian. Specifically, biased members attribute the corresponding outcome more to the DM’s decision as compared to unbiased Bayesian members. On the other hand, $\gamma _G<1$ or $\gamma _B<1$ implies that members are conservative in their response to a good or a bad signal, respectively, and attribute the corresponding outcome more to the DM’s luck as compared to unbiased Bayesian members.^27^

Finally, we can also capture asymmetric updating of beliefs, i.e., asymmetric attribution of outcomes to the DM’s decision and luck. If $\gamma _G>\gamma _B$ ($\gamma _G<\gamma _B$), then members are more likely to attribute a good (bad) outcome to the DM’s decision and a bad (good) outcome to luck.

*Estimating deviations from Bayes’ rule.* We now estimate Eq. (2) using ordinary least squares (OLS) to analyze the biases that members suffer from when updating their beliefs.^28^ Figure D1 of Appendix D.3 shows the distribution of subjects who update their beliefs inconsistently (i.e., in the opposite direction to that predicted by Bayes’ rule) or not at all. The inclusion of these observations in the analysis may result in biased or incorrect conclusions, particularly if these subjects are reporting beliefs that do not genuinely reflect their true posterior beliefs. Hence, for the remainder of the analysis, we exclude a subject if 25% or more of their posterior beliefs are inconsistent (44 out of 272 subjects in total) or if they report a posterior belief equal to the interim belief across all six rounds of the experiment (23 subjects in total). These two groups jointly constitute 24.6% of the sample. Note that these numbers are largely in line with what is found in the literature (see, e.g.,Möbius et al. 2014; Coutts 2019; Barron 2020).^29^

Table 4 presents the regression results of members both at the pooled level (column 1) and at the treatment level (columns 2 to 5). As a test of Hypothesis 2, our primary interest is to examine whether the coefficients are different from 1. Hence, asterisks are used in the table to indicate whether a coefficient is statistically significantly different from 1.

**Table 4:** Regression of members’ posterior beliefs

	Dependent variable: $\mathrm {Logit}$(posterior)				
	(1)	(2)	(3)	(4)	(5)
Variables	Pooled	RA	LA	HA	GA
$\delta$ : $\mathrm {logit}$(interim belief)	0.695***	0.764***	0.692***	0.703***	0.529***
	(0.039)	(0.071)	(0.054)	(0.058)	(0.135)
$\gamma _G$ : Good outcome $\times$ $\mathrm {logit}(p)$	0.751***	0.744***	0.622***	0.847*	0.798**
	(0.051)	(0.089)	(0.079)	(0.081)	(0.098)
$\gamma _B$ : Bad outcome $\times$ $\mathrm {logit}(1-p)$	0.966	0.932	1.058	0.946	0.876
	(0.067)	(0.092)	(0.117)	(0.072)	(0.114)
Observations	2,460	410	820	820	410
# Subjects (clusters)	205	205	205	205	205
R-squared	0.608	0.686	0.651	0.583	0.421
$$ \underline{{Test } \,\, {of} \,\, \gamma _G=\gamma _B}$$					
test statistic	−3.190	−1.588	−3.065	−1.081	−0.512
*p* value	0.002	0.114	0.002	0.281	0.609

Robust standard errors clustered at the subject level in parentheses. This analysis excludes subjects classified as inconsistent or non-updaters.

*** *p* < 0.01, ** *p* < 0.05, * *p* < 0.10. Null hypothesis is coefficient = 1.

Column (1) shows that group members are biased in their belief-updating process. The estimate for $\delta$ suggests that they suffer from base-rate neglect on average (test of $\delta =1$: *p* value < 0.001).^30^ The estimate for $\gamma _G$ suggests that after controlling for the weight members place on their interim beliefs, members are conservative in their responses to good outcomes. That is, they attribute good outcomes to luck more than a Bayesian would and this effect is statistically significant (test of $\gamma _G=1$: *p* value < 0.001). However, there is no statistically significant evidence that members respond to bad outcomes differently from a Bayesian (test of $\gamma _B=1$: *p* value = 0.608). Hence, relative to the Bayesian benchmark, group members give too little credit for the DM’s success but the right amount of blame for the DM’s failure.

The last two rows of Table 4 present the results of a Wald test of equality between $\gamma _G$ and $\gamma _B$, giving us a test of the presence of an asymmetric attribution bias. Overall, members update their beliefs about the DM asymmetrically (i.e., $\gamma _G<\gamma _B$). They tend to attribute good outcomes more to luck and, relatively, bad outcomes more to the DM’s decision. This effect is statistically significant (*p* value = 0.002).^31^

We next analyze the members’ updating behavior across the different appointment mechanisms. The coefficient estimates in columns (2)–(5) of Table 4 reveal that biases similar to the ones observed at the pooled level exist at the treatment level. Under each appointment mechanism, members consistently suffer from base-rate neglect, attribute good outcomes more to luck, and treat bad outcomes no differently from a Bayesian.^32^^,^ 33 The asymmetry observed in the attribution of outcomes is statistically significant in treatment LA only (Wald tests of $\gamma _G=\gamma _B$: *p* values = 0.002, 0.114, 0.281 and 0.609, respectively, for treatments LA, RA, HA and GA).^34^

In summary, we do not find support for Hypothesis 2. Members are not Bayesian when updating their beliefs after observing their DM’s outcomes.

##### Result 2

On average, group members exhibit similar biases in their updating behavior under all the appointment mechanisms. They suffer from base-rate neglect in their updating behavior. Compared to the Bayesian benchmark, members attribute good outcomes more to luck, but their average response to bad outcomes is not different from Bayesian.

We next consider the relationship between subjects’ effort choices (which are determined by their types) and their updating behavior. The results in Sect. 2.3.2 reveal that subjects’ effort choices are correlated with their interim beliefs. Our aim here is to test whether subjects who exert high effort as DMs update their beliefs differently to those who exert low effort after controlling for their interim beliefs.

Table 5 reports separate parameter estimates of Eq. (2) based on whether the subjects have chosen low effort (column 1) or high effort (column 2) as DMs within a given round in the investment task. The estimates of $\delta$ and $\gamma _B$ are not statistically significantly different between columns (1) and (2) (*p* values = 0.222 and 0.818, respectively). However, the estimate of $\gamma _G$ is statistically significantly different between the two columns (*p* value = 0.035). While the estimate for $\gamma _G$ is statistically significantly less than 1 in column (1) (*p* value < 0.001), it is not different from 1 in column (2) (*p* value = 0.697). Hence, regardless of their effort choices as DMs in a given round of the task, subjects suffer from base-rate neglect ($\delta <1$) and are no different from a Bayesian in their response to bad outcomes ($\gamma _B=1$) on average. However, in a given round of the investment task, those individuals who choose low effort as DMs are more likely to attribute good outcomes to luck when they make decisions as group members.^35^ This suggests that the consensus effect that we find to be driving members’ interim beliefs also appears to be shaping their updating behavior.

**Table 5:** Regression of members’ posterior beliefs based on effort choice as DMs

	Dependent variable: $\mathrm {Logit}$(posterior)	
	(1)	(2)
Variables	Chose low effort	Chose high effort
$\delta$ : $\mathrm {logit}$(interim belief)	0.710***	0.603***
	(0.048)	(0.073)
$\gamma _G$ : Good outcome $\times$ $\mathrm {logit}(p)$	0.698***	0.957
	(0.059)	(0.110)
$\gamma _B$ : Bad outcome $\times$ $\mathrm {logit}(1-p)$	0.923	0.951
	(0.080)	(0.100)
Observations	1,646	814
# subjects (clusters)	190	125
R-squared	0.626	0.553
$$ \underline{{Test } \,\, {of} \,\, \gamma _G=\gamma _B}$$		
test statistic	−2.568	0.049
*p* value	0.011	0.961

Robust standard errors clustered at the subject level in parentheses. This analysis excludes subjects classified as inconsistent or non-updaters.

*** p < 0.01, ** * p* < 0.05, * * p* < 0.10. Null hypothesis is coefficient = 1.

## Experiment 2

### Motivation and design

The results from Experiment 1 suggest that subjects use their own behavior as the basis for forming and updating their beliefs about others. We design Experiment 2 to investigate this further.

Experiment 2 consists of two treatments. In treatment S (single role), subjects are informed at the beginning of the experiment whether they have been assigned as the DM or as a group member. They make incentivized decisions only in their assigned roles. Specifically, they are asked to make their investment decisions (only if they are the DM) or to report their beliefs about the DM’s decisions (only if they are the member). After reporting their beliefs at the end of each round, members are asked to indicate, hypothetically, what their investment decision would have been if they were the DM. This structure allows us to examine the relationship between individuals’ effort choices as DMs and their beliefs as members, while mitigating the impact that experience may have on their beliefs.

The key question we ask in Experiment 2 is whether we continue to observe the consensus effect in members’ attribution of outcomes in treatment S. Given the well-documented evidence on the consensus effect in driving behavior (e.g., Ross et al., 1977; Marks & Miller, 1987; Engelmann & Strobel, 2000, 2012), we expect the relationship between subjects’ behavior and their attribution of outcomes to persist even if they do not make choices as decision makers prior to stating their beliefs.^36^ Consequently, we expect to observe similar biases in the overall attribution of outcomes when decisions are no longer elicited using the strategy method.^37^

The second treatment that we implement in Experiment 2 is treatment D (dual role), where subjects make decisions both in the roles of the DM and a group member (as in Experiment 1). Treatment D is designed to provide a comparable benchmark against which to evaluate subjects’ behavior in treatment S. This is important given the differences in format between Experiment 1 and Experiment 2, which we outline below. A total of 297 subjects (99 DMs and 198 group members) participated in treatment S, and 206 subjects (making decisions in both roles) participated in treatment D.

Unlike Experiment 1, which was conducted in a physical laboratory, the sessions for Experiment 2 were conducted online during a lockdown amid the COVID-19 pandemic.^38^ Subjects were recruited from a similar subject pool at the University of Melbourne, with between 18 and 24 subjects in each virtual session.^39^ The experiments were programmed using oTree (Chen et al., 2016). For each session, all the subjects were admitted into a Zoom meeting with their videos and microphones turned off. They were provided with separate links for the instructions to each part of the experiment, and the experimenter read out the instructions in the Zoom meeting. Each session lasted about 60 minutes on average.

The implementation of Experiment 2 was different on two other dimensions to accommodate the different and shorter nature of the online sessions. First, since we do not find any significant evidence in Experiment 1 that members’ updating behavior depends on the appointment mechanism (Result 2), we removed this treatment variation by using only the random-appointment mechanism (RA) to determine the DM of each group. Second, each group participated in only three rounds of the investment task (instead of six), remaining in the same group and role for all three rounds. In two of the rounds, we used the same parameters as in Game 0 and Game 1 from Experiment 1. We introduced Game 2 as the third set of parameters. Relative to Game 1, both investments in Game 2 provide a higher return of 900 ECU if they succeed and the same low return of 150 ECU if they fail.^40^ We randomized the order of these parameters across the groups within each session.

### Results

Table D9 in Appendix D.5 reveals that there are significant differences in the subject pools between Experiment 1 and Experiment 2. Subjects in Experiment 2 are on average slightly older, less likely to be Australian or majoring in economics, more likely to be a postgraduate student, and more experienced with economics experiments. However, with respect to the behavioral variables, the subjects do not differ in their decisions in the dictator game or risk task between Experiment 1 and Experiment 2.

We are mainly interested in analyzing members’ posterior beliefs in treatment S, to see whether the same type of biases as in Experiment 1 exist and whether we find evidence of a consensus effect.^41^ To test for the consensus effect, we use the hypothetical choices that members make when they are asked what their investment decision would have been if they were the DM. Table D10 in Appendix D.5 reveals that there is a statistically significant and positive relationship between members’ hypothetical choices as DMs and their incentivized decisions in the dictator game. Hence, the hypothetical answers given by the members seem to be indicative of their underlying preferences.

We start by analyzing the posterior beliefs elicited in Round 1 of treatment S, which provides the cleanest examination of members’ biases absent any experience in the decision-making process as DMs.^42^ Columns (1) and (2) of Table 6 present parameter estimates of Eq. (2) based on whether members indicate, hypothetically, that they would have chosen low effort or high effort, respectively, if they were the DM of the group. Columns (3) and (4) report parameter estimates including members’ belief updates in all rounds of the investment task. In the table, we also present *p* values of pairwise comparisons of parameter estimates between columns (1) and (2), and between columns (3) and (4).

**Table 6:** Regression of members’ posterior beliefs based on hypothetical effort choice as DMs (treatment S)

	Dependent variable: $\mathrm {Logit}$(posterior)					
	Round 1 only			All rounds		
	(1)	(2)	(1) vs. (2)	(3)	(4)	(3) vs. (4)
Variables	Low effort	High effort	*p* value	Low effort	High effort	*p* value
$\delta$ : $\mathrm {logit}$(prior belief)	0.560***	0.395***	0.191	0.483***	0.607***	0.254
	(0.110)	(0.062)		(0.079)	(0.077)	
$\gamma _G$ : Good outcome $\times$ $\mathrm {logit}(p)$	0.490*	0.995	0.092*	0.601*	1.068	0.085*
	(0.262)	(0.143)		(0.210)	(0.173)	
$\gamma _B$ : Bad outcome $\times$ $\mathrm {logit}(1-p)$	0.774	0.636*	0.688	1.181	0.759	0.082*
	(0.288)	(0.192)		(0.188)	(0.171)	
Observations	204	92		656	232	
# subjects (clusters)	102	46		137	71	
R-squared	0.370	0.532		0.392	0.488	

Robust standard errors clustered at the subject level in parentheses. This analysis excludes subjects classified as inconsistent or non-updaters.

All columns control for members’ hypothetical effort choices in treatment S. Columns (1) and (2) restrict the analysis to the first round of updates only.

***p < 0.01, ** p < 0.05, * *p* < 0.10. Null hypothesis is coefficient = 1.

Columns (1) and (2) reveal that members who would have chosen low effort as DMs are more likely to attribute the DM’s good outcomes to luck relative to a Bayesian (*p* value = 0.054), while those who would have chosen high effort are no different from a Bayesian in their attribution of good outcomes (*p* value = 0.972). Overall, members who would have chosen low effort are more likely to attribute good outcomes to luck than those who would have chosen high effort (*p* value = 0.092).^43^ These findings are consistent with those from Experiment 1 (Table 5). Interestingly, we now find that members who would have chosen high effort as DMs are more likely to attribute bad outcomes to luck relative to a Bayesian (*p* value = 0.064). However, there is no statistically significant difference in the attribution of bad outcomes between members who would have chosen high effort and those who would have chosen low effort (*p* value = 0.688).

Columns (3) and (4) of Table 6 reveal that, when we consider all rounds of the task, members who would have chosen low effort as DMs attribute good outcomes more to luck both relative to a Bayesian (*p* value = 0.060) and relative to those who would have chosen high effort as DMs (*p* value = 0.085). These results are consistent with those observed in columns (1) and (2), as well as Experiment 1. Moreover, we now find that members who would have chosen high effort as DMs are more likely to attribute bad outcomes to luck as compared to members who would have chosen low effort as DMs (*p* value = 0.082).^44^

Finally, we compare members’ updating behavior across treatment S, treatment D, and Experiment 1. Tables D11 and D12 in Appendix D.5 present comparisons of members’ updating behavior between treatments S and D, separately by members’ effort choices and at the pooled level, respectively. The last column of Table D12 also provides *p* values from tests of differences in parameter estimates between Experiment 1 and Experiment 2. The tables provide two main insights. First, we do not find any systematic differences between treatments S and D in members’ attribution of outcomes, both when we compare subjects separately based on their effort choices as DMs, and at the pooled level. Second, despite the differences in the experimental design, format, and subject pool, we do not find any statistically significant differences in members’ attribution of outcomes between Experiment 1 and Experiment 2.

Hence, we conclude that there is no evidence to suggest that members’ biases in their attribution of the DM’s outcomes are driven by whether or not they have experience in the decision-making process as DMs. With and without the experience of acting as a decision maker, the same attribution biases exist and seem to be driven by a consensus effect.

## Conclusion

In many environments, the determinants of outcomes are not observable. What beliefs do individuals hold in such circumstances about the determinants of others’ outcomes? Do they attribute the outcomes to luck or to the decisions made? Do the beliefs depend on the outcome, i.e., whether the outcome is good or bad? These are the questions we address in this paper.

Our results reveal that members suffer from biases in the way they attribute outcomes to luck versus the choices made. Moreover, members treat good and bad outcomes differently in the sense that while they attribute good outcomes more to luck as compared to a Bayesian, their response to bad outcomes is no different from a Bayesian. This asymmetry implies that the credit decision makers receive for good outcomes is less than the blame they get for bad outcomes.

In Experiment 1, we find that group members exhibit similar biases under all the mechanisms used to appoint the decision maker. However, we find that biases in updating behavior tend to be driven by those subjects who choose low effort as decision makers. Interestingly, the consensus effect we detect affects both initial beliefs and updating behavior. In Experiment 2, we show that the same type of biases exist even if the subjects do not have experience as decision makers.

Determining the systematic biases that individuals may have in the way they process new information and update their beliefs about the decisions of others is critical in a wide range of economic and social interactions. One general implication of our study is that the biases we identify may affect the generosity of decision makers in environments where social preferences matter. For example, they may act less generously if they know that they will not receive sufficient credit for good outcomes. The biases may also affect decision makers’ willingness to take risk. For instance, if business or political leaders are aware that they are given relatively more blame for their failures than credit for their successes, then this may perpetuate a culture of failure avoidance. Such a ‘fear of failure’ culture may reduce their incentives to exert costly effort or their tolerance towards risk.

Our study identifies the biases which exist in the evaluation of others’ decisions specifically in contexts where prosocial preferences play a key role in decision making. In future research, it would be interesting to understand whether the same type of biases exist in other contexts. For example, if performance in a skill-based task is important for leadership, do we observe that the same type of biases emerge in evaluation? Or, if we remove the anonymity of the decision maker, to what extent do in-group versus out-group considerations affect leadership evaluation? Answering these questions would broaden our understanding of biases in performance evaluation.

## Supplementary Information

Below is the link to the electronic supplementary material.

Supplementary material 1 (PDF 669 KB)

## Footnotes

1 For example, within the policy domain, redistribution decisions may be driven by beliefs about the determinants of income (e.g., Alesina & Angeletos, 2005) or self-serving attribution biases (Deffains et al., 2016). Misattribution of determinants have also been shown to affect consumer choice (Haggag et al., 2019).

2 See, for example, Kahneman and Tversky (1973), Nisbett and Borgida (1975) and Tversky and Kahneman (1982) on base rate neglect, and Lord et al. (1979), Darley and Gross (1983), Plous (1991) and Rabin and Schrag (1999) on confirmatory bias.

3 See Engelmann and Strobel (2000) for a discussion of the difference between the consensus effect and the false consensus effect. Ascribing of one’s own motivation, feelings, and behavior onto other people is also referred to as “social projection” (see, e.g., Holmes, 1968; Ames, 2004). The psychological motivations underpinning social projection may be a desire to conform or feel connected to others, or a desire to justify one’s own behavior. Social projection and the consensus effect may also be a heuristic that individuals use when making decisions in uncertain environments.

4 Experience in the decision-making process could allow members to put themselves into the shoes of the decision maker. Such perspective taking may influence their behavior and biases (see, e.g., Galinsky & Moskowitz, 2000; Todd et al., 2011; Lange et al., 2021).

5 Asymmetries in information processing have been analyzed and found in other domains as well. For example, Garrett and Sharot (2017) and Sunstein et al. (2017) show how good news and bad news are treated differently in the context of different life events and climate change, respectively. Evidence also suggests that individuals may treat new information that is in line with their convictions and new information that is against their expectations asymmetrically. For instance, Nyhan and Reifler (2010) analyze this in the context of political news and Sharot et al. (2011) study this with respect to health outcomes.

6 See also Palfrey and Wang (2012), who study the responsiveness of prices to signals in asset markets.

7 The instructions can be found in Appendix A.1.

8 This setup resembles modern performance evaluation practices used by many organizations, such as 360-degree feedback, where decision makers are evaluated both by their colleagues (who may have similar responsibilities to them) and their subordinates (whose outcomes may be affected by the decision makers’ actions) (see, e.g., Lepsinger & Lucia, 2009).

9 Subjects are given the instructions for the investment task after they have made their decisions in the dictator game. This ensures that any strategic behavior in the dictator game is minimized. The actual decisions of subjects in the dictator game are not observed by their group members during the experiment. In the investment task, subjects are only informed about the mechanism that would be used to appoint the DM in each round.

10 They are paid an additional 10 ECU if both of their guesses are correct.

11 See, e.g., Galeotti and Zizzo (2018), for a similar protocol.

12 Previous studies have found that subjects perform better in terms of Bayesian updating and additivity when beliefs are elicited as a population frequency. Gigerenzer and Hoffrage (1995), for example, find that subjects are more capable of performing Bayesian updating when probabilities are presented in the form of frequencies. Price (1998) finds that subjects are less likely to report extreme values in their beliefs when the questions are framed as relative frequencies. Schlag et al. (2015) argue that these findings point to the advantage of eliciting beliefs as frequencies rather than as probabilities.

13 We used two separate screens. Screenshots of the decision screens can be found in Appendix B.

14 Specifically, for a given belief report $r\in [0,100]$, the group member receives 10 ECU with probability $1-\left[ I(e=e_H)-\frac{r}{100}\right] ^2$, where $I(e=e_H)$ is an indicator variable that equals 1 if the DM chose $e_H$ (Investment X) and 0 otherwise.

15 To avoid cultural factors influencing behaviour (Alesina & Angeletos, 2005; Rey-Biel et al., 2018), only Australian citizens were recruited. For eight subjects, we observed a mismatch between their citizen information on the recruitment system and their response relating to length of stay in the country in the post-experiment questionnaire. Further, two subjects had prior experience with the experiment. Hence, data from 272 subjects are used for the main analysis. Our results however are robust to the inclusion of the eight (non-Australian) subjects.

16 For example, a cell that states “RA1” means that the DM was appointed randomly for that round (treatment RA), and the subjects played Game 1.

17 As indicated in Fig. 1, subjects were informed that in previous experiments, (i) about 80% of participants transferred a positive amount to their matched partner, and (ii) for those who transferred, the average transfer was about 40% of their endowment. These statistics were obtained using data from pilot experiments ($N=192$).

18 See Rotemberg (2014) for a survey of models of social preferences used in the literature. Brock et al. (2013), Cappelen et al. (2013), and Exley (2015) analyze social preferences under risk. We differ from these papers with our focus on how the group members evaluate the outcomes of the DMs in risky environments.

19 Note that $$\frac{Q_H}{N}+\omega -c_L> \frac{Q_L}{N}+\omega -c_L = \frac{Q_H}{N}+\omega -c_H > \frac{Q_L}{N}+\omega -c_H$$.

20 For instance, under the assumption of risk neutrality, $\beta ^*=\frac{1}{2}$.

21 Hence, data from treatments LA0 and LA1 are pooled together as treatment LA, while data from treatments HA0 and HA1 are pooled together as treatment HA.

22 Interestingly, there is a higher proportion of individuals who transfer nothing to their matched partner in the dictator game, but who choose high effort as DMs in treatment HA compared to the other treatments. This may be because these individuals believe that they are unlikely to be appointed as the DM in this treatment and therefore think that their effort choice is less likely to be implemented.

23 We also elicited members’ incentivized beliefs about their DM’s behavior in the dictator game under each appointment mechanism. We find that there is a positive relationship between group members’ interim beliefs and their reports of how much the DM has transferred in the dictator game. This further shows that the subjects regard the dictator game as a predictor of an individual’s likelihood of choosing high effort as a DM.

24 Pairwise comparisons provide initial tests of Hypothesis 1. The Kolmogorov-Smirnov tests reveal that the distributional differences are statistically significant in all comparisons (RA vs. LA: *p* value < 0.001; RA vs. HA: *p* value < 0.001; GA vs. HA: *p* value < 0.001) except one (RA vs. GA: *p* value = 0.664). Similarly, Wilcoxon signed-rank tests reject the null hypotheses that the average interim belief is equal between treatments RA and LA (*p* value < 0.001), RA and HA (*p* value < 0.001), RA and GA (*p* value = 0.006) and GA and HA (*p* value < 0.001).

25 Consistent with this finding, the consensus effect is also present in members’ beliefs about the DM’s behavior in the dictator game. Specifically, in additional regression analyses of members’ beliefs about the DM’s dictator game behavior, we find a statistically significant positive relationship between the members’ own giving behavior and their beliefs about the DM’s giving behavior in the dictator game.

26 See, e.g., Grether (1980), Möbius et al. (2014), Ambuehl and Li (2018), Buser et al. (2018), and Coutts (2019) for similar estimation approaches.

27 Note that, as explained in Benjamin (2019, p. 103), the existence and direction of one type of bias does not imply anything about the existence and direction of the other type of bias. For example, an individual can put too little weight on their interim belief and be conservative in their response to signals at the same time.

28 Note that the $\mathrm {logit}$ function is only defined for beliefs in (0,100). Instead of excluding observations of subjects who state 0 or 100 as their interim or posterior belief about the DM, we take the $\mathrm {logit}$ of 0.01 or 99.99 as an approximation. Also, one potential concern with estimating (2) using OLS is that the estimates are biased if there are measurement errors in the subjects’ reported beliefs. For example, subjects could make mistakes or are imprecise when reporting their beliefs. For robustness, we also consider an alternative specification where the appointment mechanisms are used as instruments for the logit of members’ interim beliefs for the analysis at the pooled level. This instrumental-variable (IV) approach requires that the appointment mechanisms are a strong predictor of the members’ interim beliefs (as we show in Sect. 2.3.2) and do not have a separate direct effect on their posterior beliefs. We find that the IV estimates lead to similar conclusions. Details of the results from the IV regression analysis can be found in Appendix D.2.

29 We present the analyses including these subjects in Table D5 of Appendix D.3. The main results remain robust despite an attenuation of the coefficient estimates of $\gamma _G$ and $\gamma _B$. Tables D6 and D7 of Appendix D.3 also reveal that our results are robust to using different criteria for excluding inconsistent updaters and non-updaters.

30 An alternative and less restrictive specification to (2) is to allow $\delta$ (the weight placed on interim beliefs) to vary with the signal received by the members. However, estimating $\delta _G$ and $\delta _B$ separately, we find that both of these parameter estimates are statistically significantly different from 1 (*p* values < 0.001 for both), but the difference between them is not statistically significant (*p* value = 0.692). Moreover, this less restrictive specification does not affect the estimates of $\gamma _G$ and $\gamma _B$.

31 We also consider heterogeneity in updating behavior using finite mixture model analyses in Appendix D.4. The results show that the majority of belief updates in the sample is characterized by base-rate neglect, under-responsiveness to DMs’ outcomes, and an asymmetric attribution of DMs’ outcomes to decision and luck.

32 Note that base-rate neglect and under-inference are commonly observed in other studies adopting a similar estimation strategy to our paper (Benjamin, 2019). However, we do not observe under-inference relative to a Bayesian in response to bad outcomes.

33 Comparing the magnitudes of the biases across the appointment mechanisms, we fail to reject the null hypotheses that the estimates for $\delta$, $\gamma _G$, and $\gamma _B$ are jointly equal to one another (Wald tests of $\delta ^{RA}=\delta ^{LA}=\delta ^{HA}=\delta ^{GA}$: *p* value = 0.395; $\gamma _G^{RA}=\gamma _G^{LA}=\gamma _G^{HA}=\gamma _G^{GA}$: *p* value = 0.110; and $\gamma _B^{RA}=\gamma _B^{LA}=\gamma _B^{HA}=\gamma _B^{GA}$: *p* value = 0.686).

34 Note that the magnitude of the asymmetry is the largest in treatment LA, which is driven both by the estimate for $\gamma _G$ being the lowest and that for $\gamma _B$ being the highest in this treatment. This suggests that across the four treatments, group members are the least likely to believe that good outcomes result from a choice of high effort in the case when the most selfish individual is appointed to be the DM.

35 Our results here complement those of Di Tella et al. (2015) who show that individuals avoid altruistic actions by distorting their beliefs about others’ altruism.

36 Even though we do not elicit incentivized effort choices from members, a consensus effect may still exist if members consider in their minds what their actions would have been as DMs before stating their beliefs.

37 Brandts and Charness (2011) show that the strategy method typically finds qualitatively similar effects compared to the direct-response method.

38 The same experimenter ran both Experiment 1 and Experiment 2. Instructions for Experiment 2 are available in Appendix D.2. Experiment 2 (the design, treatments, empirical strategy, and power calculations) is pre-registered on the AEA RCT Registry (AEARCTR-0006519: https://doi.org/10.1257/rct.6519-1.1).

39 Unlike Experiment 1, both domestic and international students were recruited for the sessions in Experiment 2.

40 Our theoretical prediction is that DMs are more likely to choose high effort in Game 2 as compared to in Game 1.

41 Note that in Experiment 2, we consider members’ unconditional beliefs as *prior* beliefs since, unlike Experiment 1, there are no variations in the appointment mechanisms that would have otherwise influenced the members’ unconditional beliefs.

42 While we only report results on members’ updating behavior here, we also examine the DMs’ effort choices and members’ prior beliefs in Experiment 2. Our results are consistent with our findings from Experiment 1. Specifically, there exists a positive relationship between the DM’s decision to choose high effort in the investment task and their giving behavior in the dictator game. Moreover, using members’ hypothetical choices as DMs, we continue to find evidence of a consensus effect. On average, members who would have chosen high effort as DMs have higher prior beliefs about the DM choosing high effort.

43 We show later that the results in Experiment 2 have similar patterns as those in Experiment 1. The noisier behaviour of subjects in the virtual environment, however, may have led to some estimates being less precise in Experiment 2.

44 Note that this result is consistent with a consensus effect being present also for those members who would have chosen high effort as DMs.
