# Supplementary material for: By chance or by choice? Biased attribution of others’ outcomes when social preferences matter
Source: Exp Econ. 2021 Sep 28;25(2):413–43. doi: 10.1007/s10683-021-09731-w (PMC8477728; doi:10.1007/s10683-021-09731-w)
Supplement: Supplementary file 1 — Supplementary material 1 (PDF 669 KB) [file 10683_2021_9731_MOESM1_ESM.pdf]

[FOR ONLINE PUBLICATION]

A Experimental instructions

A.1 Experiment 1 (Lab)

Overview of Experiment

Thank you for agreeing to take part in this study which is funded by the Australian Research Council. Please read the following instructions carefully. A clear understanding of the instructions will help you make better decisions and increase your earnings from the experiment.

You will participate in two experiments today: Experiment 1 and Experiment 2.[1] You will receive detailed instructions for each experiment before you participate in them. Note that your decisions in Experiment 2 will not change the earnings that you receive from Experiment 1. You will be informed of the outcomes of both experiments at the end of today's session.

You will be paid for the decisions you make in either Experiment 1 or Experiment 2. This implies that you should carefully consider all of the decisions you make in both experiments as they may determine your earnings. Whether you will be paid for Experiment 1 or Experiment 2 will be randomly determined at the end of the session. Your final payment today will also include a $10 participation fee.

During the experiments, we will be using Experimental Currency Units (ECU). At the end of the session, we will convert the amount you earn into Australian Dollars (AUD) using the following conversion rate: 10 ECU = 1 AUD.

At the end of Experiment 2, you will be asked to fill out a brief questionnaire asking you some general questions. All of the decisions you make in today's session will remain anonymous.

Please do not talk to one another during the experiment. If you have any questions, please raise your hand and we will come over to answer your questions privately.

[1] Note that in the instructions, we use Experiment 1 and Experiment 2 to refer to the two tasks (dictator game and investment task) that subjects participate in.

Experiment 1

You will participate in Experiment 1 in groups of two. The computer will randomly match you with one other person in the room. You will never learn the identity of your partner.

Each of you is given an endowment of 300 ECU, and you are asked to divide this amount between yourself and the person you are matched with.

At the end of today's session, if this experiment is picked for payment, then you will be paid either according to your decision or according to the decision made by your randomly matched partner. The computer will randomly determine whose allocation decision will be implemented.

Example. Suppose you choose to divide your endowment by keeping 200 ECU for yourself and giving 100 ECU to your matched partner. Your matched partner decides to keep 130 ECU and give 170 ECU to you. If, at the end of the experiment, the computer randomly determines that it is the allocation of your matched partner that gets implemented, then your payment will be 170 ECU and your matched partner's payment will be 130 ECU.

Are there any questions? If not, we will proceed with Experiment 1.

Experiment 2

Experiment 2 consists of six identical rounds. At the end of the experiment, if you are paid for Experiment 2, then the computer will randomly pick one of the six rounds for payment.

You will participate in each round in groups of three. At the beginning of each round, the computer will randomly match you with two other people in this room with whom you have not been matched before. You will never learn the identity of your partners. Each round consists of three stages.

Stage 1: Appointment of a group leader.

In this stage, one group member will be assigned to be the leader of the group. There will be four possible methods to determine who is assigned the role of the leader. At the beginning of each round, the computer will reveal which method will be used to determine the leader for that round.

Method 1: One group member will be randomly assigned by the computer to be the leader. Hence, each group member has an equal chance of being assigned the role of the leader.

Method 2: The group member who transferred the lowest amount to his/her matched partner in Experiment 1 will be assigned to be the leader (ties will be broken randomly).

Method 3: The group member who transferred the highest amount to his/her matched partner in Experiment 1 will be assigned to be the leader (ties will be broken randomly).

Method 4: Each individual within the group will be asked to indicate whether you prefer your leader to be someone who has transferred the highest or the lowest amount to his/her matched partner in Experiment 1. The computer will then randomly pick one of the decisions of the group members to implement. If your decision is implemented, then one of your other two group members will be appointed to be the leader based on your preference. Hence, you will not be appointed to be the leader if your decision is implemented.

Example 1. Suppose the leader is appointed using Method 4. In Experiment 1, Player 1 chose to transfer 100 ECU to his/her matched partner, and Player 2 chose to transfer 160 ECU to his/her matched partner. Player 3 indicates that his/her preferred leader is someone who has transferred the lowest amount to his/her matched partner in Experiment 1. If the computer randomly determines that Player 3's decision will be implemented, then Player 1 will be assigned the role of the leader.

You will only need to indicate your preferred leader for Method 4 once, at the beginning of Experiment 2. The same decision will be used whenever Method 4 is being used to determine the appointment of the group leader.

Stage 2: Investment decision by the group leader.

The leader will be given an endowment of 300 ECU. S/he will be asked to choose between two investment options that will affect the payoffs of all group members. Each investment can either fail or succeed. The two investment options have different chances of success/failure. They also have different costs to the leader.

Specifically, the two investments are:

Investment X: This investment costs 250 ECU to the leader. It will succeed with a 75% chance, and fail with a 25% chance.

Investment Y: This investment costs 50 ECU to the leader. It will succeed with a 25% chance, and fail with a 75% chance.

The payoffs to the leader and each group member in this stage of Experiment 2 are calculated as follows:

1. Payoff to leader = 300 ECU − Cost of investment + Returns on investment
2. Payoff to each group member = Returns on investment

Note that the amount that you receive from each investment may be different in each round, and this may affect the final payoffs to the leader and each group member. However, you will always receive a higher payoff if the investment succeeds, and a lower payoff if it fails. Please pay attention to these numbers on the screen in each round.

Figure A1 shows an example where the returns of each investment options are 200 ECU if the investment succeeds, and 0 ECU if the investment fails, i.e., as shown by the numbers in **red**.

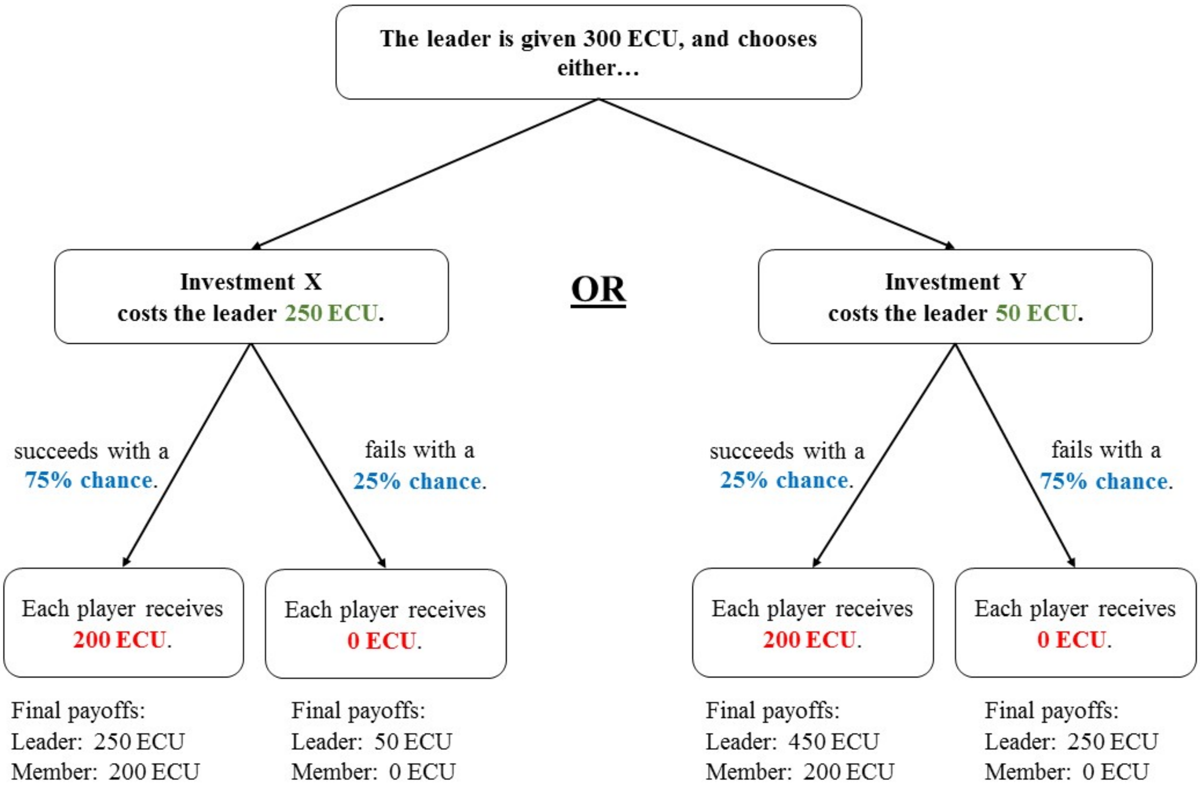

Figure A1: Investment Options (Example of a Round)

Example 2. Suppose in the round depicted in Figure A1, the leader chooses Investment X for the group. Then, the investment costs the leader 250 ECU, and will succeed with a 75% chance and fail with a 25% chance. At the end of the experiment, if the investment succeeds, then each group member will receive 200 ECU, and the leader will receive $(300 - 250 + 200) = 250$ ECU for this stage of Experiment 2.

Example 3. Suppose in the round depicted in Figure A1, the leader chooses Investment Y for the group. Then, the investment costs the leader 50 ECU, and will succeed with a 25% chance and fail with a 75% chance. At the end of the experiment, if the investment fails, then each group member will receive 0 ECU, and the leader will receive $(300 - 50 + 0) = 250$ ECU for this stage of Experiment 2.

You will be informed whether you have been assigned the role of the leader at the end of the experiment. Hence, you will be asked to make an investment decision in Stage 2 of each round assuming that you have been assigned the role of the leader. Your decision will be implemented if you have been assigned the role of the leader for that round.

At the end of the experiment, all group members will learn how much they have received from the chosen investment, but they will not learn the investment decision of the leader.

Stage 3: Beliefs of the other group members.

After you have made your investment decision, you will be asked to predict which investment your leader has chosen, assuming that someone else in your group has been assigned the role of the leader.

Specifically, we would like to know how likely it is in your opinion that the leader has chosen Investment X. *Suppose there were 100 people in the position the leader is in now. How many of them do you think would choose Investment X?*

You will need to choose a number between 0 and 100. A higher number means that you think the leader is more likely to have chosen Investment X.

The specific questions you will be asked are listed below.

Question 1

Suppose there were 100 people in the position the leader is in now. How many of them do you think would choose Investment X?

In Question 2, you are given additional information. You are asked to evaluate the same question with this additional information. Specifically, you should consider whether your guess of the leader's decision will be different, given that you know the outcome of the investment chosen by your leader.

Question 2

> **Suppose you are informed that the investment chosen by your leader has succeeded, and you have therefore received the high payoff.**
>
> Now consider whether your guess will be higher than, lower than, or the same as the one you stated in Question 1. That is, suppose there were 100 people in the position the leader is in now. Given an outcome of high payoff, how many of them do you think have chosen Investment X?
>
> **Suppose you are informed that the investment chosen by your leader has failed, and you have therefore received the low payoff.**
>
> Now consider whether your guess will be higher than, lower than, or the same as the one you stated in Question 1. That is, suppose there were 100 people in the position the leader is in now. Given an outcome of low payoff, how many of them do you think have chosen Investment X?

The computer will randomly select one of these two questions and you will be paid for your response to this question. If Question 2 is chosen for payment, then you will be paid for your answer to the scenario that corresponds to the actual outcome of the investment chosen by your leader.

The section below describes how your payoff in Stage 3 will be determined. This procedure has been used in many other studies. We explain the procedure in detail, but what is most important is that this payoff structure is designed such that it is in your best interest to report your true belief about your leader's decision.

Your payment for the question randomly chosen by the computer is determined as follows. You will receive 10 ECU with some chance. Your chance of receiving 10 ECU depends on your answer and the leader's decision. The closer your guess is to the actual decision made by your leader, the higher is your chance of receiving the fixed payment of 10 ECU.

Specifically, your chance of receiving 10 ECU is determined by the following formula:

$$\text{Chance of receiving 10 ECU} = \left[1 - \left(\frac{x - \text{your guess}}{100}\right)^2\right] \times 100.$$

x takes the value of 100 if your leader chose Investment X, and x takes the value of 0 if your leader chose Investment Y.

To illustrate, suppose your leader has chosen Investment X. This means that $x = 100$ in the formula above, and your chance of receiving 10 ECU will be higher if your guess is higher. If you state 100 as your guess that the leader has chosen Investment X, then your chance of receiving 10 ECU will be $[1 - (\frac{100-100}{100})^2] \times 100 = 100$. On the other hand, suppose your leader has chosen Investment Y instead, while your guess remains at 100. This means that $x = 0$ in the formula above, and your chance of receiving 10 ECU will be $[1 - (\frac{0-100}{100})^2] \times 100 = 0$.

Here is another example:

Example 4. Suppose you guess 70 as the chance that your leader has chosen Investment X for the group. At the end of the experiment, the computer reveals that your leader has chosen Investment X for the group. Hence, your chance of receiving 10 ECU will be $[1 - (\frac{100-70}{100})^2] \times 100 = 91$.

To determine whether you receive 10 ECU, the computer will randomly draw a number between 0 and 100 (including decimal points). If the number drawn by the computer is less than or equal to your chance of receiving 10 ECU as determined by the formula above, then you will receive 10 ECU. Otherwise, you will receive 0 ECU. Hence, in Example 4 above, if the number randomly drawn by the computer is less than or equal to 91, then you will receive 10 ECU. Otherwise, you will receive 0 ECU.

Payment for Experiment 2:

At the end of the experiment, if you are paid for Experiment 2, then the computer will randomly select one of the six rounds for payment. For the randomly chosen round:

1. If you are assigned the role of the leader, then you will be paid according to your investment decision in Stage 2 only.
2. If you are not assigned the role of the leader, then you will be paid according to your leader's investment decision in Stage 2, plus your decisions in Stage 3. The computer will randomly select one of the two questions in Stage 3, and you will be paid for your response to this question.

Summary

1. You will participate in six identical rounds in Experiment 2. At the beginning of each round, the computer will randomly match you to a new group with two other people. Each round consists of three stages.

2. In Stage 1, one group member will be assigned to be the leader of the group. There are four possible methods to determine who is assigned the role of the leader. You will be informed which method will be used to determine the leader at the beginning of each round.

   In Method 1, the computer will randomly assign one group member to be the leader.

   In Method 2, the group member who transferred the lowest amount to his/her matched partner in Experiment 1 will be assigned to be the leader.

   In Method 3, the group member who transferred the highest amount to his/her matched partner in Experiment 1 will be assigned to be the leader.

   In Method 4, you will be asked to indicate whether you prefer your leader to be someone who has transferred the highest or the lowest amount to his/her matched partner in Experiment 1. The computer will pick one of the decisions of the group members to implement. If your decision is implemented, then one of your other two group members will be appointed to be the leader based on your preference. Hence, you will not be appointed to be the leader if your decision is implemented.

   You will be asked to indicate your preferred leader for Method 4 once, at the beginning of Experiment 2. The computer will use the same decision whenever Method 4 is being used to determine the leader.

3. In Stage 2, you will be asked to make an investment decision, assuming that you have been assigned the role of the leader. The leader will be given an endowment of 300 ECU, and s/he will be asked to choose between two investment options that will affect the payoffs of all group members. Your decision will be implemented for your group only if you have been assigned the role of the leader for that round.

4. Investment X and Investment Y may be different in each round. In each round, the amount that you receive from each investment may be different, but you will always receive a higher payoff if the investment succeeds, and a lower payoff if it fails. The investment options will be shown on your computer screens.

5. In Stage 3, you will be asked to predict which investment your leader has chosen, assuming that you have not been assigned the role of the leader. You will be asked two questions.

   In Question 1, you will be asked to predict how likely it is in your opinion that the leader has chosen Investment X. You will need to choose a number between 0 and 100. A higher number means that you think the leader is more likely to have chosen Investment X.

   In Question 2, you are given additional information. Specifically, you will be asked the same question under two different scenarios: (i) suppose you are told that the investment has succeeded; and (ii) suppose you are told that the investment has failed. You should consider whether your guess of the leader's decision will be higher than, lower than, or the same as the one you stated in Question 1, given that you know the outcome of the investment chosen by your leader.

6. The payoff structure used to determine your payment in Stage 3 is designed such that it is in your best interest to report your true beliefs about your leader's decision.

7. At the end of the experiment, the computer will randomly select one of the six rounds for payment. For the randomly chosen round, if you are assigned the role of the leader, then you will be paid according to your decision in Stage 2. If you are not assigned the role of the leader, then you will be paid according to your leader's decision in Stage 2, as well as your decisions in Stage 3. The computer will randomly select one of the two questions in Stage 3 for payment.

If you have any questions, please raise your hand and an experimenter will come to you to answer your questions privately. Otherwise, please wait patiently for the experimenter to launch the practice questions on your computer screens. The purpose of these practice questions is to make sure that you understand the experiment. If you have any questions at any time, please raise your hand and an experimenter will come over to answer your questions privately.

Once everyone has completed the practice questions, we will proceed with one practice round for Experiment 2. The purpose of the practice round is to allow you to familiarize yourself with the decision screens. Your decisions in the practice round will not affect your payments for today's experiment. We will proceed with Experiment 2 once everyone has completed the practice round.

Practice Questions (Experiment 2)

1. I will be paid for the decisions in both experiments today. True/False [Ans: False]

2. We will participate in six identical rounds in Experiment 2. If we are paid for Experiment 2, then we will be paid for our decisions in one of the six rounds. True/False. [Ans: True]

3. We will participate in each round of Experiment 2 in groups of three. One group member will be assigned the role of the leader. True/False [Ans: True]

4. In Experiment 1, Player 1 chose to transfer 160 ECU to his/her matched partner, Player 2 chose to transfer 115 ECU to his/her matched partner, and Player 3 chose to transfer 160 ECU to his/her matched partner.

   Suppose the leader is appointed using Method 2. Which of the following is correct? [Ans: (b)]

   (a) Player 1 will be assigned the role of the leader.
   (b) Player 2 will be assigned the role of the leader.
   (c) Player 3 will be assigned the role of the leader.
   (d) Both Player 1 and Player 3 have an equal chance of being assigned the role of the leader.

5. In the above example, suppose the leader is appointed using Method 3. Which of the following is correct? [Ans: (d)]

   (a) Player 1 will be assigned the role of the leader.
   (b) Player 2 will be assigned the role of the leader.
   (c) Player 3 will be assigned the role of the leader.
   (d) Both Player 1 and Player 3 have an equal chance of being assigned the role of the leader.

6. Suppose the leader is appointed using Method 4. Suppose also that your preference for leadership appointment is randomly chosen by the computer to be implemented. Which of the following is correct? [Ans: (b)]

   (a) Depending on what I indicate as my preference of the appointed leader, I have a chance of being assigned the role of the leader.
   (b) Regardless of what I indicate as my preference of the appointed leader, I will definitely not be assigned the role of the leader.

7. Suppose the leader is appointed using Method 4. In Experiment 1, Player 1 chose to transfer 200 ECU to his/her matched partner, and Player 2 chose to transfer 85 ECU to his/her matched partner. Player 3 indicates that his/her preferred leader is someone who has transferred the highest amount to his/her matched partner in Experiment 1.

   Suppose Player 3's decision is randomly chosen by the computer to be implemented. Which of the following is correct? [Ans: (a)]

   (a) Player 1 will be assigned the role of the leader.
   (b) Player 2 will be assigned the role of the leader.
   (c) Both Player 1 and Player 2 have an equal chance of being assigned the role of the leader.

8. Which of the following is correct? [Ans: (b)]

   (a) The other group members will be informed of the investment chosen by the leader, but not the amount they have received from the investment.
   (b) The other group members will be informed of the amount they have received from the investment chosen by the leader, but not the investment chosen by him/her.
   (c) The other group members will be informed of the investment chosen by the leader, and the amount they have received from the investment.

9. Consider the investment options depicted in the figure below.

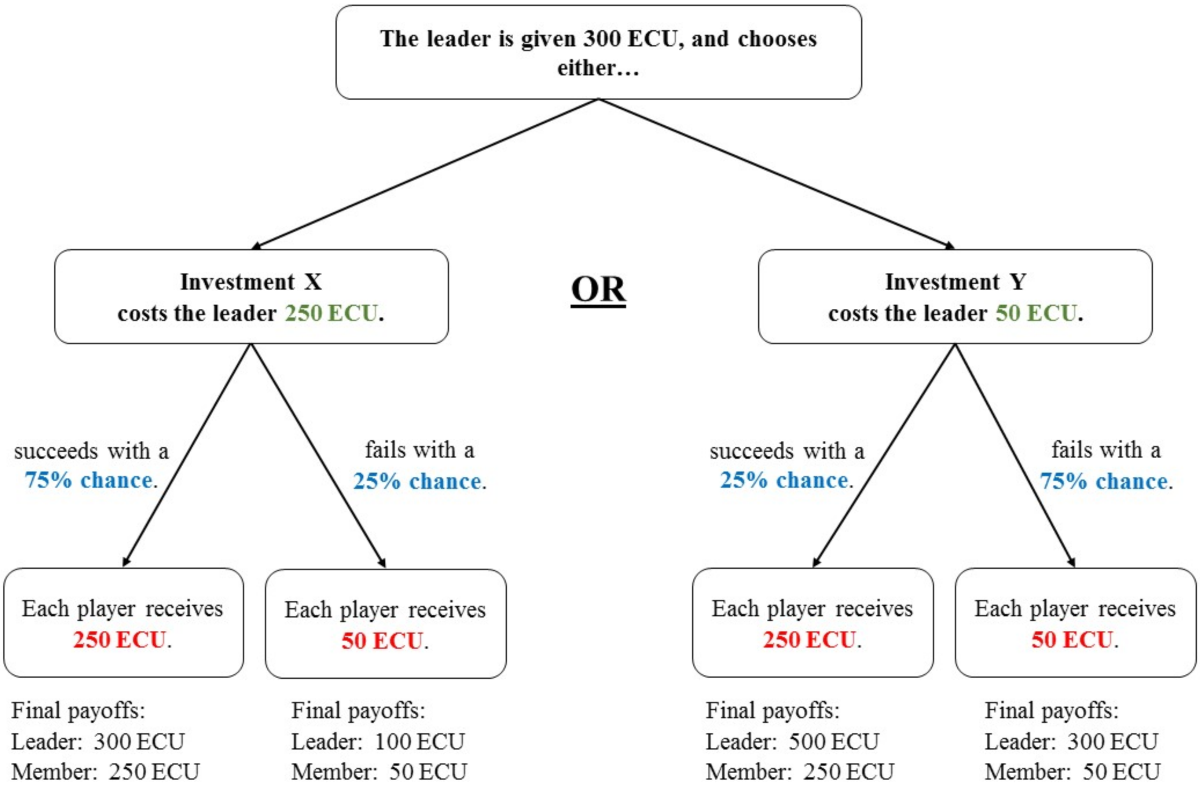

Figure A2: Investment Options (Practice Question)

Suppose the leader chooses Investment X.

(a) At the end of the experiment, the computer randomly determines that the investment succeeds.

If you are not the leader, how many ECU will you receive from Stage 2 of Experiment 2? [Ans: 250 ECU]

(b) At the end of the experiment, the computer randomly determines that the investment fails.

If you are the leader, how many ECU will you receive from Stage 2 of Experiment 2? [Ans: 100 ECU]

10. Which of the following is true? [Ans: (c)]
    (a) I will be paid for my decision in Stage 3 of Experiment 2 regardless of whether I have been assigned the role of the leader or not.
    (b) I will be paid for my decision in Stage 3 of Experiment 2 only if I have been assigned the role of the leader.
    (c) I will be paid for my decision in Stage 3 of Experiment 2 only if I have not been assigned the role of the leader.

11. In Stage 3, I will be asked two questions. If I am paid for Stage 3 of Experiment 2, then I will be paid according to my answers to both questions. True/False [Ans: False]

12. Suppose you strongly believe that the leader of your group has chosen Investment Y. Which of the following statement is true? [Ans: (b)]
    (a) It is in my best interest to choose a higher number as my guess of "how likely is my leader to have chosen Investment X".
    (b) It is in my best interest to choose a lower number as my guess of "how likely is my leader to have chosen Investment X".
    (c) It is in my best interest to choose 50 as my guess of "how likely is my leader to have chosen Investment X".

A.2 Experiment 2 (Online)

Overview

Thank you for participating! You will receive \$5 for completing today's experiment, and the instructions explain how you can make decisions and earn more money.

You will participate in two tasks today: Task 1 and Task 2. You will be paid for the decisions you make in either Task 1 or Task 2. Whether you will be paid for Task 1 or Task 2 will be randomly determined by the computer at the end of the session.

During the experiments, we will be using Experimental Currency Units (ECU). At the end of the session, we will convert the amount you earn into Australian Dollars (AUD) using the following conversion rate: 20 ECU = 1 AUD.

At the end of Task 2, you will be asked to fill out a brief questionnaire asking you some general questions. All of the decisions you make in today's session will remain anonymous, and you will never learn the identities of the other participants in today's session.

Task 1

(Treatment S)

You will participate in Task 1 in groups of three. The computer will randomly match you with two other people from today's experiment. You will stay in the same group for all of Task 1.

You will participate in three rounds of a decision task. Each round consists of two stages.

Stage 1: Investment decision by the group leader.

One group member will be randomly assigned by the computer to be the leader at the beginning of Task 1. In Stage 1, if you are the leader, you will be asked to make an investment decision for your group. Your role will remain the same for all three rounds of Task 1.

If you are the leader, you will be given an endowment of 300 ECU in each round. You will choose between two investment options that will affect both your payoff and the other group members' payoffs. Each investment can either fail or succeed. The two investment options have different chances of success/failure, as well as different costs to you.

Specifically, the two investment options are:

Investment X: This investment costs you 250 ECU. It will succeed with a 75% chance and fail with a 25% chance.

Investment Y: This investment costs you 50 ECU. It will succeed with a 25% chance and fail with a 75% chance.

The payoffs to the leader and each group member in this stage are calculated as follows:

1. Payoff to leader = 300 ECU − Cost of investment + Returns on investment
2. Payoff to each group member = Returns on investment

The table below shows an example where the return from each investment option is 200 ECU if it succeeds, and 0 ECU if it fails. These values are shown in **red**.

Investment Option	Cost to Leader	Investment		Payoff to each Member if investment:		Payoff to Leader if investment:	
		Succeeds	Fails	Succeeds	Fails	Succeeds	Fails
X	250	75%	25%	200	0	250 (= 300 − 250 + 200)	50 (= 300 − 250 + 0)
Y	50	25%	75%	200	0	450 (= 300 − 50 + 200)	250 (= 300 − 50 + 0)

Table A1: Investment Options (Example of a Round)

Example. Suppose you are the leader, and you choose Investment X as shown in Table 1 above. Then, the investment costs you 250 ECU, and it will succeed with a 75% chance and fail with a 25% chance. If the investment succeeds, then you will receive (300 − 250 + 200) = 250 ECU and each of your group members will receive 200 ECU. Instead if the investment fails, then you will receive (300 − 250 + 0) = 50 ECU and each of your group members will receive 0 ECU.

Please note that in each round of Task 1, the returns from the investment options will be different. Within each round, both investments will provide the same high return if they succeed, and the same low return if they fail. However, the chance of failure and success will be different for different options (as stated above). Please pay attention to these values on the screen.

The other group members will never learn your investment decisions in the three rounds. At the end of the experiment, they will learn how much they will receive from the chosen investment, but they will not learn whether you chose Investment X or Y.

Stage 2: Beliefs of the other group members.

In Stage 2, if you are not assigned to be the leader of your group, you will be asked to predict how likely it is in your opinion that your leader has chosen Investment X.

The specific questions you will be asked are listed below.

Question 1

How likely do you think it is that your Leader has chosen Investment X? Specifically, what is the chance out of 100 that s/he has chosen Investment X?

In Question 2, you are given additional information. You are asked to evaluate the same question with this additional information.

Question 2(a)

Suppose you are informed that the investment chosen by your leader has succeeded. This gives you a high payoff.
Now consider whether your prediction will be higher than, lower than, or the same as the one you stated in Question 1. Specifically, given that the investment has succeeded, what is the chance out of 100 that s/he has chosen Investment X?

Question 2(b)

Suppose you are informed that the investment chosen by your leader has failed. This gives you a low payoff.
Now consider whether your prediction will be higher than, lower than, or the same as the one you stated in Question 1. Specifically, given that the investment has failed, what is the chance out of 100 that s/he has chosen Investment X?

For both questions, you will need to choose a number between 0 and 100. A higher number means that you think your leader is more likely to have chosen Investment X.

For your payment, the computer will randomly select one of these two questions and you will be paid for your response to this question. If Question 2 is chosen for payment, you will be paid for Question 2(a) if the investment has succeeded or Question 2(b) if it has failed.

To determine your payment, we use a procedure which has been used in many other studies. For the question randomly chosen by the computer, you receive either 200 ECU or 0 ECU. The closer your prediction is to the actual decision made by your leader, the higher is your chance of receiving 200 ECU. Hence, what is most important is that this procedure is designed such that it is in your best interest to report your true belief about the chance that your leader has chosen Investment X.

[The exact details of how your payment will be determined are available here (link to separate document) if you are interested, but it is not necessary for you to read these notes.]

Payment for Task 1:

At the end of the experiment, if you are paid for Task 1, then the computer will randomly select one of the three rounds for payment. For the randomly chosen round:

1. **If you are the leader**, then you will be paid according to your investment decision in Stage 1 only.
2. **If you are not the leader**, then you will be paid either according to your leader's investment decision in Stage 1, or your predictions in Stage 2, but not both. The computer will randomly determine which one you will be paid for.

Summary

1. In Task 1, the computer will randomly divide you into groups of three. One group member will be randomly assigned by the computer to be the leader of the group. You will stay in the same group and role for all of Task 1. You will be informed whether you are assigned to be the leader at the beginning of Task 1.
2. You will participate in three rounds in Task 1. Each round consists of two stages.
3. In Stage 1, if you are the leader, you will be asked to make an investment decision. You will be given an endowment of 300 ECU in each round, and you will choose between two investment options that will affect both your payoffs and the other group members' payoffs.
4. The returns from Investment X and Investment Y will be different in each round. However, within each round, both investments always provide the same high return if they succeed and the same low return if they fail.
5. In Stage 2, if you are not the leader, you will be asked to predict how likely it is in your opinion that your leader has chosen Investment X. The payoff structure used to determine your payment in Stage 2 is designed such that it is in your best interest to report your true belief about your leader's decision.
6. At the end of the experiment, the computer will randomly select one of the three rounds for payment. For the randomly chosen round:
   (a) If you are the leader, then you will be paid according to your decision in Stage 1.
   (b) If you are not the leader, then you will be paid either according to your leader's decision in Stage 1, or your predictions of your leader's decision in Stage 2.

You have arrived at the end of the instructions for Task 1.

Please return to the experiment and click the button on the screen to start the practice questions.

Task 1

(Treatment D)

You will participate in Task 1 in groups of three. The computer will randomly match you with two other people from today's experiment. You will stay in the same group for all of Task 1.

You will participate in three rounds of a decision task. Each round consists of two stages.

Stage 1: Investment decision by the group leader.

One group member will be randomly assigned by the computer to be the leader at the beginning of Task 1. You will be informed of your role at the end of the experiment. In Stage 1, you will be asked to make an investment decision assuming that you are the leader of your group. Your decision will be implemented at the end of the experiment if you are the leader of your group. Your role will remain the same for all three rounds of Task 1.

If you are the leader, you will be given an endowment of 300 ECU in each round. You will choose between two investment options that will affect both your payoff and the other group members' payoffs. Each investment can either fail or succeed. The two investment options have different chances of success/failure, as well as different costs to you.

Specifically, the two investment options are:

> Investment X: This investment costs you 250 ECU. It will succeed with a 75% chance and fail with a 25% chance.
>
> Investment Y: This investment costs you 50 ECU. It will succeed with a 25% chance and fail with a 75% chance.

The payoffs to the leader and each group member in this stage are calculated as follows:

1. Payoff to leader = 300 ECU − Cost of investment + Returns on investment
2. Payoff to each group member = Returns on investment

The table below shows an example where the return from each investment option is 200 ECU if it succeeds, and 0 ECU if it fails. These values are shown in **red**.

Investment Option	Cost to Leader	Investment		Payoff to each Member if investment:		Payoff to Leader if investment:	
		Succeeds	Fails	Succeeds	Fails	Succeeds	Fails
X	250	75%	25%	200	0	250 (= 300 − 250 + 200)	50 (= 300 − 250 + 0)
Y	50	25%	75%	200	0	450 (= 300 − 50 + 200)	250 (= 300 − 50 + 0)

Table A2: Investment Options (Example of a Round)

Example. Suppose you are the leader, and you choose Investment X as shown in Table 1 above. Then, the investment costs you 250 ECU, and it will succeed with a 75% chance and fail with a 25% chance. If the investment succeeds, then you will receive (300 − 250 + 200) = 250 ECU and each of your group members will receive 200 ECU. Instead if the investment fails, then you will receive (300 − 250 + 0) = 50 ECU and each of your group members will receive 0 ECU.

Please note that in each round of Task 1, the returns from the investment options will be different. Within each round, both investments will provide the same high return if they succeed, and the same low return if they fail. However, the chance of failure and success will be different for different options (as stated above). Please pay attention to these values on the screen.

If, at the end of the experiment, your decisions are implemented as the leader of your group, the other group members will never learn your investment decisions in the three rounds. At the end of the experiment, they will learn how much they will receive from the chosen investment, but they will not learn whether you chose Investment X or Y.

Stage 2: Beliefs of the other group members.

In Stage 2, you will be asked to predict how likely it is in your opinion that the leader has chosen Investment X, assuming that someone else in your group has been assigned the role of the leader.

The specific questions you will be asked are listed below.

Question 1

How likely do you think it is that your Leader has chosen Investment X? Specifically, what is the chance out of 100 that s/he has chosen Investment X?

In Question 2, you are given additional information. You are asked to evaluate the same question with this additional information.

Question 2(a)

Suppose you are informed that the investment chosen by your leader has succeeded. This gives you a high payoff.
Now consider whether your prediction will be higher than, lower than, or the same as the one you stated in Question 1. Specifically, given that the investment has succeeded, what is the chance out of 100 that s/he has chosen Investment X?

Question 2(b)

Suppose you are informed that the investment chosen by your leader has failed. This gives you a low payoff.
Now consider whether your prediction will be higher than, lower than, or the same as the one you stated in Question 1. Specifically, given that the investment has failed, what is the chance out of 100 that s/he has chosen Investment X?

For both questions, you will need to choose a number between 0 and 100. A higher number means that you think your leader is more likely to have chosen Investment X.

For your payment, the computer will randomly select one of these two questions and you will be paid for your response to this question. If Question 2 is chosen for payment, you will be paid for Question 2(a) if the investment has succeeded or Question 2(b) if it has failed.

To determine your payment, we use a procedure which has been used in many other studies. For the question randomly chosen by the computer, you receive either 200 ECU or 0 ECU. The closer your prediction is to the actual decision made by your leader, the higher is your chance of receiving 200 ECU. Hence, what is most important is that this procedure is designed such that it is in your best interest to report your true belief about the chance that your leader has chosen Investment X.

[The exact details of how your payment will be determined are available here (link to separate document) if you are interested, but it is not necessary for you to read these notes.]

Payment for Task 1:

At the end of the experiment, if you are paid for Task 1, then the computer will randomly select one of the three rounds for payment. For the randomly chosen round:

1. **If you are the leader**, then you will be paid according to your investment decision in Stage 1 only.
2. **If you are not the leader**, then you will be paid either according to your leader's investment decision in Stage 1, or your predictions in Stage 2, but not both. The computer will randomly determine which one you will be paid for.

Summary

1. In Task 1, the computer will randomly divide you into groups of three. One group member will be randomly assigned by the computer to be the leader of the group. You will stay in the same group and role for all of Task 1. You will be informed whether you are assigned to be the leader at the end of the experiment.
2. You will participate in three rounds in Task 1. Each round consists of two stages.
3. In Stage 1, you will be asked to make an investment decision, assuming that you are the leader. You will be given an endowment of 300 ECU in each round, and you will choose between two investment options that will affect both your payoffs and the other group members' payoffs. At the end of the experiment, your decision will be implemented for your group only if you are the leader of your group.
4. The returns from Investment X and Investment Y will be different in each round. However, within each round, both investments always provide the same high return if they succeed and the same low return if they fail.
5. In Stage 2, you will be asked to predict how likely it is in your opinion that your leader has chosen Investment X, assuming that someone else in your group has been assigned the role of the leader. The payoff structure used to determine your payment in Stage 2 is designed such that it is in your best interest to report your true belief about your leader's decision.
6. At the end of the experiment, the computer will randomly select one of the three rounds for payment. For the randomly chosen round:
   (a) If you are the leader, then you will be paid according to your decision in Stage 1.
   (b) If you are not the leader, then you will be paid either according to your leader's decision in Stage 1, or your predictions of your leader's decision in Stage 2.

You have arrived at the end of the instructions for Task 1.

Please return to the experiment and click the button on the screen to start the practice questions.

Task 2

You will participate in Task 2 in groups of two. The computer will randomly match you with one other person in today's session.

Each of you is given an endowment of 300 ECU, and you are asked to divide this amount between yourself and the person you are matched with.

At the end of today's session, if Task 2 is picked for payment, then you will be paid either according to your decision or according to the decision made by your randomly matched partner. The computer will randomly determine whose allocation decision will be implemented.

Example. Suppose you choose to divide your endowment by keeping 200 ECU for yourself and giving 100 ECU to your matched partner. Your matched partner decides to keep 130 ECU and give 170 ECU to you. If, at the end of the experiment, the computer randomly determines that it is the allocation of your matched partner that gets implemented, then your payment will be 170 ECU and your matched partner's payment will be 130 ECU.

Are there any questions? If not, we will proceed with Task 2.

Practice Questions (Task 1)

(Treatments S and D)

Note: Each question is shown on a separate screen on oTree.

1. I will be paid for the decisions in both tasks today. True/False [Ans: False]
2. Task 1 has three rounds. If Task 1 is chosen for payment, then I will be paid for the decisions in one of the three rounds. True/False [Ans: True]
3. (Treatment S) We will participate in Task 1 in groups of three. One group member will be assigned the role of the leader. I will remain in the same group and role for all rounds of Task 1. True/False [Ans: True]

   (Treatment D) We will participate in Task 1 in groups of three. One group member will be assigned the role of the leader. I will remain in the same group for all rounds of Task 1. True/False [Ans: True]
4. Which of the following is correct? [Ans: (b)]
   (a) The other group members will be informed of the investment chosen by the leader, but not the amount they have received from the investment.
   (b) The other group members will be informed of the amount they have received from the investment chosen by the leader, but not the investment chosen by him/her.
   (c) The other group members will be informed of the investment chosen by the leader, and the amount they have received from the investment.
5. Consider the investment options given in the table below.

Investment Option	Cost to Leader	Investment		Payoff to each Member if investment:		Payoff to Leader if investment:	
		Succeeds	Fails	Succeeds	Fails	Succeeds	Fails
X	250	75%	25%	250	50	300 (= 300 − 250 + 250)	100 (= 300 − 250 + 50)
Y	50	25%	75%	250	50	500 (= 300 − 50 + 250)	300 (= 300 − 50 + 50)

Suppose the leader chooses **Investment X**.

At the end of the experiment, the computer randomly determines that the investment **succeeds**.

If you are **not the leader**, how many ECU will you receive from Stage 1? [Ans: 250]

6. Consider the investment options given in the table below.

Investment Option	Cost to Leader	Investment		Payoff to each Member if investment:		Payoff to Leader if investment:	
		Succeeds	Fails	Succeeds	Fails	Succeeds	Fails
X	250	75%	25%	250	50	300 (= 300 − 250 + 250)	100 (= 300 − 250 + 50)
Y	50	25%	75%	250	50	500 (= 300 − 50 + 250)	300 (= 300 − 50 + 50)

Suppose the leader chooses **Investment X**.

At the end of the experiment, the computer randomly determines that the investment **fails**.

If you are **the leader**, how many ECU will you receive from Stage 1? [Ans: 100]

7. Which of the following is true? [Ans: (c)]
   (a) If I am the leader, then I will be paid for my decisions in Stage 2.
   (b) If I am not the leader, then I will be paid for BOTH my leader's decision in Stage 1 AND my decisions in Stage 2.
   (c) If I am not the leader, then I will be paid for EITHER my leader's decision in Stage 1 OR my decisions in Stage 2.

8. If I am not the leader and I am paid for Stage 2, then I will be paid according to my answers to both Question 1 and Question 2. True/False [Ans: False]

9. (Treatment S) Suppose you strongly believe that the leader of your group has chosen Investment Y.
   (Treatment D) Suppose you are not the leader. You strongly believe that the leader of your group has chosen Investment Y.
   Which of the following statement is true? [Ans: (b)]
   (a) It is in my best interest to choose a high number as my prediction of the chance that my leader has chosen investment X.
   (b) It is in my best interest to choose a low number as my prediction of the chance that my leader has chosen investment X.
   (c) It is in my best interest to choose 50 as my prediction of the chance that my leader has chosen investment X.

B Screenshots for belief elicitation task in Experiment 1

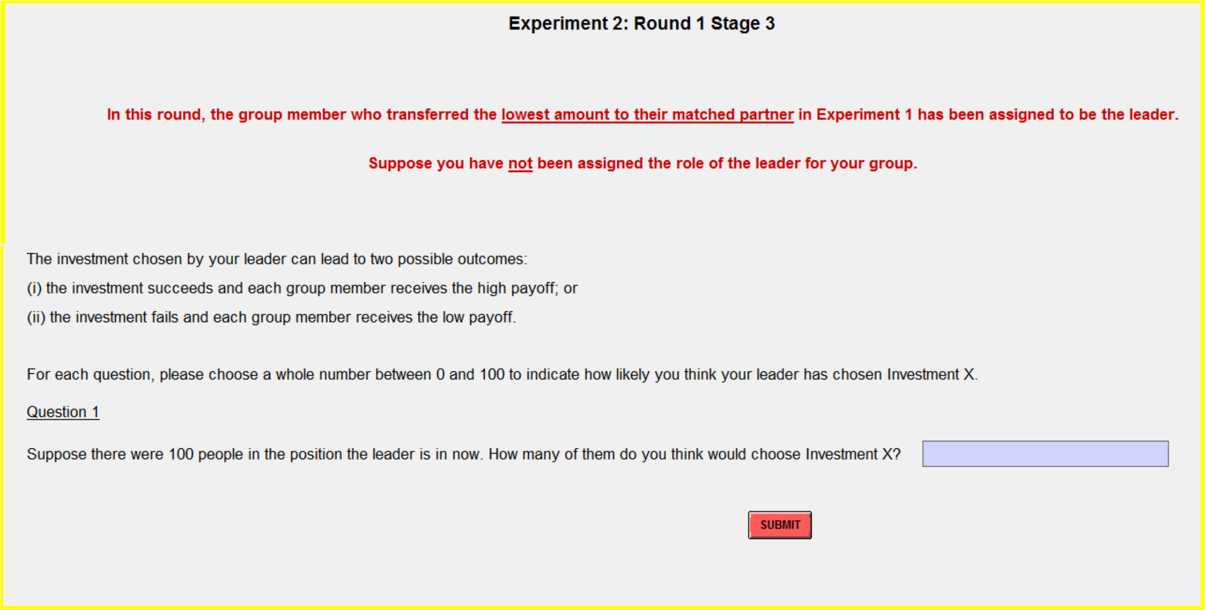

Experiment 2: Round 1 Stage 3

In this round, the group member who transferred the <u>lowest amount to their matched partner</u> in Experiment 1 has been assigned to be the leader.

Suppose you have <u>not</u> been assigned the role of the leader for your group.

The investment chosen by your leader can lead to two possible outcomes:
(i) the investment succeeds and each group member receives the high payoff; or
(ii) the investment fails and each group member receives the low payoff.

For each question, please choose a whole number between 0 and 100 to indicate how likely you think your leader has chosen Investment X.

<u>Question 1</u>

Suppose there were 100 people in the position the leader is in now. How many of them do you think would choose Investment X?

SUBMIT

(a) Interim Belief

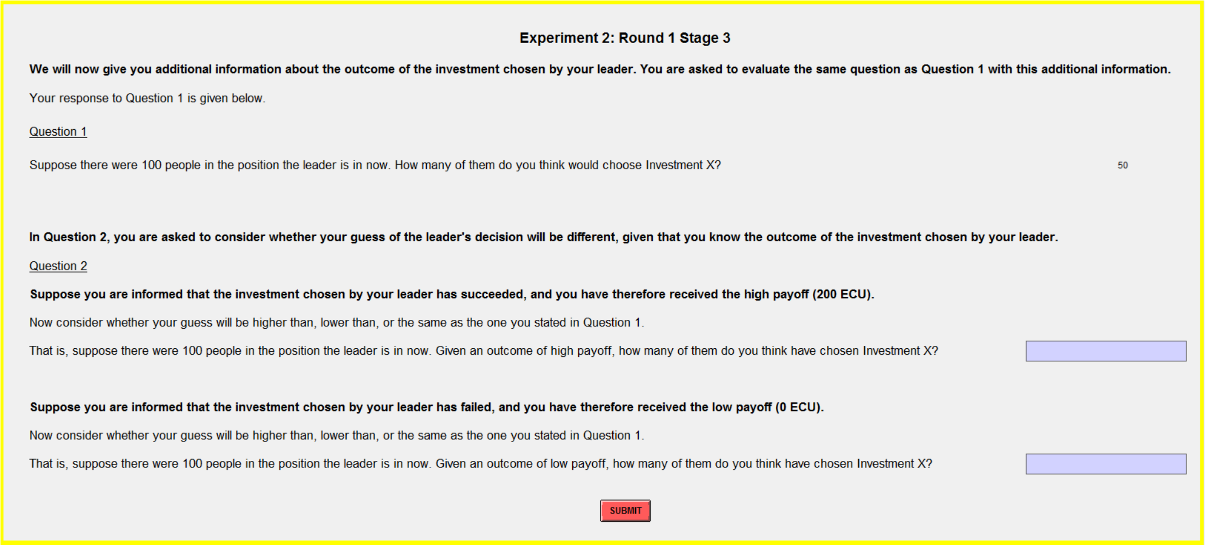

Experiment 2: Round 1 Stage 3

We will now give you additional information about the outcome of the investment chosen by your leader. You are asked to evaluate the same question as Question 1 with this additional information.

Your response to Question 1 is given below.

<u>Question 1</u>

Suppose there were 100 people in the position the leader is in now. How many of them do you think would choose Investment X? 50

In Question 2, you are asked to consider whether your guess of the leader's decision will be different, given that you know the outcome of the investment chosen by your leader.

<u>Question 2</u>

Suppose you are informed that the investment chosen by your leader has succeeded, and you have therefore received the high payoff (200 ECU).

Now consider whether your guess will be higher than, lower than, or the same as the one you stated in Question 1.

That is, suppose there were 100 people in the position the leader is in now. Given an outcome of high payoff, how many of them do you think have chosen Investment X?

Suppose you are informed that the investment chosen by your leader has failed, and you have therefore received the low payoff (0 ECU).

Now consider whether your guess will be higher than, lower than, or the same as the one you stated in Question 1.

That is, suppose there were 100 people in the position the leader is in now. Given an outcome of low payoff, how many of them do you think have chosen Investment X?

SUBMIT

(b) Posterior Beliefs

Figure B1: Decision screens – Elicitation of beliefs

C Derivation of Hypothesis 1

A member of the group is appointed to be the DM under one of four possible appointment mechanisms, $\Psi \in \{RA, LA, HA, GA\}$. We are interested in how members form interim beliefs about their DM's type under each appointment mechanism, given that they have been informed that someone else in the group is the DM.

Random appointment (RA). Each member has an equal chance of being appointed as the DM. This implies that

$$\mu_i^{RA} = \Pr(\beta \geq \beta^*) = 1 - F(\beta^*). \tag{C1}$$

Appointment of lowest type (LA). The member with the lowest β is appointed to be the DM. Consider member i of type β_i who is informed that someone else in the group has been appointed to be the DM under this mechanism. Hence, s/he knows that the DM has type $\beta \leq \beta_i$ as otherwise s/he would have been appointed to be the DM. Denote the minimum of the remaining $N-1$ members' types as $\beta^{\min}$.

Given this, there are two possible cases. First, if $\beta_i < \beta^*$, then it must be that $\mu_i^{LA} = 0$ since the DM has type $\beta \leq \beta_i < \beta^*$. Second, if $\beta_i \geq \beta^*$, then the probability that the appointed DM is of type $\beta \geq \beta^*$ is given by

$$\begin{aligned}
\Pr(\beta^{\min} \geq \beta^* | \beta^{\min} < \beta_i) &= 1 - \Pr(\beta^{\min} < \beta^* | \beta^{\min} < \beta_i) \\
&= 1 - \frac{\Pr(\beta^{\min} < \beta^*)}{\Pr(\beta^{\min} < \beta_i)} \quad (\text{since } \beta^* \leq \beta_i) \\
&= 1 - \frac{1 - [1 - F(\beta^*)]^{N-1}}{1 - [1 - F(\beta_i)]^{N-1}}.
\end{aligned}$$

Hence, for member i,

$$\mu_i^{LA} = \begin{cases} 0 & \text{if } \beta_i < \beta^*, \\ \frac{[1-F(\beta^*)]^{N-1} - [1-F(\beta_i)]^{N-1}}{1-[1-F(\beta_i)]^{N-1}} & \text{if } \beta_i \geq \beta^*. \end{cases} \tag{C2}$$

Clearly $\mu_i^{LA} \leq \mu_i^{RA}$ for $\beta_i < \beta^*$, which holds as an equality if $\beta^* = 1$. For $\beta_i \geq \beta^*$, $\mu_i^{LA} - \mu_i^{RA} = \frac{[1-F(\beta^*)]^{N-1} - [1-F(\beta_i)]^{N-1} - [1-F(\beta^*)]\{1-[1-F(\beta_i)]^{N-1}\}}{1-[1-F(\beta_i)]^{N-1}}$. The denominator is ≥ 0. The numerator can be simplified to give $[1-F(\beta^*)]^{N-1} - [1-F(\beta^*)] - F(\beta^*)[1-F(\beta_i)]^{N-1}$, which is ≤ 0 since $[1-F(\beta^*)]^{N-1} \leq [1-F(\beta^*)]$. Hence, $\mu_i^{LA} \leq \mu_i^{RA}$ for $\beta_i \geq \beta^*$.

Appointment of highest type (HA). The individual with the highest β is appointed to be the DM. Consider member i of type β_i who is informed that someone else in the group has been appointed to be the DM under this mechanism. Hence, s/he knows that

the DM has type $\beta \geq \beta_i$ as otherwise s/he would have been appointed as the DM. Denote the maximum of the remaining $N-1$ members' types as $\beta^{\max}$.

Given this, there are two possible cases. First, if $\beta_i \geq \beta^*$, then it must be that $\mu_i^{HA} = 1$ since the DM is of type $\beta \geq \beta_i \geq \beta^*$. Second, if $\beta_i < \beta^*$, then the probability that the appointed DM is of type $\beta \geq \beta^*$ is given by

$$\begin{aligned}\Pr(\beta^{\max} \geq \beta^* | \beta^{\max} \geq \beta_i) &= \frac{\Pr(\beta^{\max} \geq \beta^*)}{\Pr(\beta^{\max} \geq \beta_i)} \quad (\text{since } \beta^* > \beta_i) \\ &= \frac{1 - F(\beta^*)^{N-1}}{1 - F(\beta_i)^{N-1}}.\end{aligned}$$

Hence, for member i,

$$\mu_i^{HA} = \begin{cases} 1 & \text{if } \beta_i \geq \beta^*, \\ \frac{1-F(\beta^*)^{N-1}}{1-F(\beta_i)^{N-1}} & \text{if } \beta_i < \beta^*. \end{cases} \tag{C3}$$

Clearly $\mu_i^{HA} \geq \mu_i^{RA}$ for $\beta_i \geq \beta^*$, which holds as an equality if $\beta^* = 0$. For $\beta_i < \beta^*$, $\mu_i^{HA} - \mu_i^{RA} \geq 0$ since $1 - F(\beta^*)^{N-1} \geq 1 - F(\beta^*)$ and $1 - F(\beta_i)^{N-1} \leq 1$. Hence, $\mu_i^{HA} \geq \mu_i^{RA}$ for $\beta_i < \beta^*$ also.

Group appointment (GA). All members indicate how they would like their DM to be appointed. Specifically, they may choose to appoint as DM: (i) the lowest-type member; (ii) the highest-type member; or (iii) a randomly picked member. One of the group members' appointment decisions is randomly chosen to be implemented and the DM is appointed from the remaining group members based on this individual's preference.

It is trivial to see that all members will prefer to have the highest type appointed as the DM regardless of their own type. Intuitively, this is because it increases the chance that the appointed DM is of type $\beta \geq \beta^*$ and chooses a high effort level, leading to higher expected payoffs for the members.

Consider member i of type β_i who is informed that someone else in the group has been appointed to be the DM. There are two possible cases. First, if member i's appointment decision is implemented, then the probability that the DM is of type $\beta \geq \beta^*$ depends on the probability that at least one of the other $N-1$ group members is of type $\geq \beta^*$. This is given by $1 - F(\beta^*)^{N-1}$. Second, if member i's appointment decision is not implemented, then s/he knows that the DM is of type $\beta \geq \beta_i$ as otherwise s/he would have been appointed to be the DM. Specifically, the DM's type is given by the maximum of the remaining $N-2$ members' types (excluding member i and the member whose decision is implemented). The derivation of the probability that the DM is of type $\beta \geq \beta^*$ under this scenario is similar to that of mechanism HA with $N-2$ other members.

We next evaluate member i's posterior belief that his/her appointment decision has been implemented, given the information that someone else in the group has been ap-

pointed to be the DM. Using Bayes' rule, we get $\frac{\frac{1}{N}\cdot(1)}{\frac{1}{N}\cdot(1)+\frac{N-1}{N}[1-F(\beta_i)^{N-2}]} = \frac{1}{1+(N-1)[1-F(\beta_i)^{N-2}]}$. The numerator is the product of the prior probability that member i's appointment decision is implemented ($\frac{1}{N}$) and the probability that s/he is not assigned to be the DM conditional on having his/her decision implemented. Conditional on member i's decision being implemented, s/he does not become the DM with certainty. The denominator is the probability that member i is not appointed to be the DM. The first term is the same as the numerator. The second term is the product of the prior probability that member i's appointment decision is not implemented ($\frac{N-1}{N}$) and the probability that s/he is not assigned to be the DM conditional on not having his/her appointment decision implemented. Conditional on member i's appointment decision not being implemented, the probability that member i is not appointed to be the DM is $1 - F(\beta_i)^{N-2}$. This is the probability that at least someone else in the group (other than both member i and the member whose decision is implemented) has type $\beta \geq \beta_i$ and is therefore appointed to be the DM.

Putting all these together, we have for member i,

$$\mu_i^{GA} = A \times \left[1 - F(\beta^*)^{N-1}\right] + (1 - A) \times \begin{cases} 1 & \text{if } \beta_i \geq \beta^*, \\ \frac{1-F(\beta^*)^{N-2}}{1-F(\beta_i)^{N-2}} & \text{if } \beta_i < \beta^*, \end{cases} \tag{C4}$$

where $A \equiv \frac{1}{1+(N-1)[1-F(\beta_i)^{N-2}]}$.

We would like to show that $\mu_i^{GA} \geq \mu_i^{RA}$. Note that μ_i^{GA} is a convex combination of two terms since $A \leq 1$. For both $\beta_i \geq \beta^*$ and $\beta_i < \beta^*$, these two terms are $\geq \mu_i^{RA}$ for $\beta^* > 0$. For $\beta^* = 0$, $\mu_i^{GA} = \mu_i^{RA}$.

Next, we would like to show that $\mu_i^{GA} \leq \mu_i^{HA}$. Again, since μ_i^{GA} is a convex combination of two terms, it is sufficient to show that these two terms are $\leq \mu_i^{HA}$. This is clearly the case for $\beta_i \geq \beta^*$. For $\beta_i < \beta^*$, we need to show that $\frac{1-F(\beta^*)^{N-2}}{1-F(\beta_i)^{N-2}} \leq \frac{1-F(\beta^*)^{N-1}}{1-F(\beta_i)^{N-1}}$. This is equivalent to showing that

$$\frac{[1 - F(\beta^*)^{N-1}][1 - F(\beta_i)^{N-2}] - [1 - F(\beta^*)^{N-2}][1 - F(\beta_i)^{N-1}]}{[1 - F(\beta_i)^{N-2}][1 - F(\beta_i)^{N-1}]} \geq 0.$$

The denominator is ≥ 0. Let $x \equiv F(\beta^*)$ and $y \equiv F(\beta_i)$ with $x > y$ since $\beta^* > \beta_i$. Then, the numerator becomes $(1 - x^{N-1})(1 - y^{N-2}) - (1 - x^{N-2})(1 - y^{N-1})$. Simplifying gives us

$$x^{N-2} - x^{N-1} + y^{N-1} - y^{N-2} + x^{N-1}y^{N-2} - x^{N-2}y^{N-1} \tag{C5}$$

Hence, for the numerator to be ≥ 0, we need to show the following:

Claim: $x^{N-2} - x^{N-1} + y^{N-1} - y^{N-2} + x^{N-1}y^{N-2} - x^{N-2}y^{N-1} \geq 0$ for $x, y \in [0, 1]$, $x > y$, and $N > 2$.

Proof. The proof is by induction. Let $x \equiv \alpha y$, $\alpha > 1$. Then, (C5) becomes

$$(\alpha y)^{N-2} - (\alpha y)^{N-1} + y^{N-1} - y^{N-2} + \alpha^{N-1} y^{2N-3} - \alpha^{N-2} y^{2N-3} \tag{C6}$$

Consider first $N = 3$. (C6) becomes

$$\alpha y - (\alpha y)^2 + y^2 - y + \alpha^2 y^3 - \alpha y^3 = y(\alpha - 1)(1 - y)(1 - \alpha y)$$

which is ≥ 0 since $y \in [0, 1]$, $\alpha y = x \in [0, 1]$, and $\alpha > 1$. Now suppose (C6) ≥ 0 for some $N = k$. Rearranging (C6), we have

$$\alpha^{k-2} y^{k-2}(1 - \alpha y) + \alpha^{k-2} y^{2k-3}(\alpha - 1) \geq y^{k-2} - y^{k-1}. \tag{C7}$$

Next consider $N = k+1$. (C6) becomes $\alpha^{k-1} y^{k-1}(1-\alpha y) + y^k - y^{k-1} + \alpha^{k-1} y^{2k-1}(\alpha - 1)$, which is equal to

$$y \left[\alpha^{k-1} y^{k-2}(1 - \alpha y) + \alpha^{k-1} y^{2k-2}(\alpha - 1) + y^{k-1} - y^{k-2}\right]. \tag{C8}$$

We want to show that this expression is ≥ 0 given that (C7) holds. Since $y \geq 0$, this is equivalent to showing the terms inside the brackets are ≥ 0, or

$$\alpha^{k-1} y^{k-2}(1 - \alpha y) + \alpha^{k-1} y^{2k-2}(\alpha - 1) \geq y^{k-2} - y^{k-1}. \tag{C9}$$

Note that the RHS of (C9) is the same as the RHS of (C7) and is ≥ 0. To conclude the proof, we show that the LHS of (C9) is $\geq$ the LHS of (C7). Note that this is equivalent to showing

$$(\alpha - 1)(1 - \alpha y)\alpha^{k-2}(y^{k-2} - y^{(k-2)+(k-1)}) \geq 0,$$

which holds because $\alpha > 1$, $\alpha y \in [0, 1]$, and $y^{k-2} \geq y^{(k-2)+(k-1)}$. Hence, (C8) is ≥ 0 if (C7) holds. ∎

D Additional analysis

D.1 Analysis with Game 1 treatments only

This section presents the analyses with the Game 1 treatments only. We show that Results 1 and 2 hold with the exclusion of the Game 0 treatments.

Table D1 presents marginal-effects estimates from a probit model for the relationship between the subjects' decisions as DMs in the investment task and their dictator game behavior. The estimates in the table reveal that a DM who transfers 1% more of their endowment to their matched partner in the dictator game is 0.4% more likely to choose e_H in the investment task on average, and this effect is statistically significant (p-value < 0.001). Hence, we conclude that the dictator game is a good proxy for an individual's type β_i even when we consider only the Game 1 treatments.

Table D1: Regression of DM's effort choice (Game 1)

	Dependent variable: =1 if DM chooses e_H
Variables	(1)
% endowment transferred in DG	0.004***
	(0.001)
% endowment invested in RT	−0.001
	(0.001)
Treatment LA	−0.045
	(0.028)
Treatment HA	0.048
	(0.030)
Treatment GA	0.039
	(0.028)
Order Effects	Y
Observations	1,088
# subjects (clusters)	272

Marginal effects of probit model reported. Robust standard errors in parentheses. Standard errors are clustered at the subject level.
DG: Dictator Game; RT: Risk Task.
*** p<0.01, ** p<0.05, * p<0.10.

Table D2 presents OLS estimates for the regressions of interim beliefs against the treatment variables. Similar to the main analysis in the paper, we control for order effects in columns (1) and (3) and individual fixed effects in columns (2) and (4). Treatment RA

is the comparison group in all the specifications. Overall, the coefficient estimates reveal that Result 1 is robust to the exclusion of the Game 0 treatments. In particular, group members respond to the appointment mechanism in their interim beliefs in the Game 1 treatments.

Table D2: Regression of members' interim belief (Game 1)

	Dependent variable: Interim belief			
Variables	(1)	(2)	(3)	(4)
Treatment LA	−13.074***	−13.074***	−11.989***	−12.375***
	(1.484)	(1.482)	(1.465)	(1.425)
Treatment HA	9.787***	9.787***	8.702***	9.088***
	(1.397)	(1.396)	(1.352)	(1.332)
Treatment GA	2.717**	2.717**	1.813	2.135*
	(1.355)	(1.354)	(1.277)	(1.265)
Chooses high effort as DM			24.584***	15.832***
			(1.960)	(1.984)
% endowment invested in RT	−0.086*		−0.055	
	(0.045)		(0.039)	
Constant	55.525***	45.938***	45.400***	41.572***
	(3.990)	(0.812)	(3.636)	(0.890)
Order Effects	Y	N	Y	N
Individual FE	N	Y	N	Y
Observations	1,088	1,088	1,088	1,088
# subjects (clusters)	272	272	272	272
R-squared	0.111	0.233	0.278	0.306
Test of HA = GA				
test statistic	5.202	5.209	5.341	5.461
p-value	< 0.001	< 0.001	< 0.001	< 0.001

Robust standard errors clustered at the subject level in parentheses. For all regressions, treatment RA is the reference treatment.
RT: Risk Task.
*** p<0.01, ** p<0.05, * p<0.10.

Table D3 presents the results from the OLS estimation of equation (2). Similar to the main analysis in the paper, we drop the inconsistent and non-updaters in the analysis. We find that Result 2 is also robust to the exclusion of the Game 0 treatments. Within the Game 1 treatments, members suffer from base-rate neglect relative to a Bayesian (test of $\delta = 1$: p-value < 0.001), attribute good outcomes more to luck than a Bayesian would

(test of $\gamma_G = 1$: p-value < 0.001), and treat bad outcomes like a Bayesian (test of $\gamma_B = 1$: p-value = 0.492). Consequently, they tend to attribute good (bad) outcomes more to the DM's luck (decision), i.e., $\gamma_G < \gamma_B$ (p-value = 0.012). While members exhibit similar biases in their updating behavior across all the appointment mechanisms, unlike Table 4, the asymmetry in the attribution of outcomes is now marginally statistically insignificant in treatment LA (p-value = 0.103) and statistically significant in treatment HA (p-value = 0.028).

Table D3: Regression of members' posterior beliefs (Game 1)

	Dependent variable: Logit(posterior)				
	(1)	(2)	(3)	(4)	(5)
Variables	Pooled	RA	LA	HA	GA
δ : logit(interim belief)	0.733***	0.764***	0.793***	0.771**	0.529***
	(0.049)	(0.071)	(0.057)	(0.093)	(0.135)
γ_G : Good outcome × logit(p)	0.742***	0.744***	0.728***	0.752***	0.798**
	(0.060)	(0.089)	(0.078)	(0.094)	(0.098)
γ_B : Bad outcome × logit($1-p$)	0.948	0.932	0.937	0.994	0.876
	(0.076)	(0.092)	(0.119)	(0.090)	(0.114)
Observations	1,640	410	410	410	410
# subjects (clusters)	205	205	205	205	205
R-squared	0.636	0.686	0.741	0.613	0.421
Test of $\gamma_G = \gamma_B$					
test statistic	−2.522	−1.588	−1.637	−2.218	−0.512
p-value	0.012	0.114	0.103	0.028	0.609

Robust standard errors clustered at the subject level in parentheses.
This analysis includes only the Game 1 treatments but includes subjects classified as inconsistent or non-updaters.
*** p<0.01, ** p<0.05, * p<0.10. Null hypothesis is coefficient = 1.

D.2 IV regression of posterior beliefs

Table D4 presents the results from the IV estimation of equation (2). We use the appointment mechanisms as instruments for the logit of members' interim beliefs.[2] The conclusions from the IV estimates are similar to those obtained from the OLS estimates in column (1) of Table 4. Specifically, we find that members suffer from base-rate neglect relative to a Bayesian (test of $\delta = 1$: p-value < 0.001). Moreover, members attribute good outcomes more to luck than a Bayesian would (test of $\gamma_G = 1$: p-value < 0.001), but they are no different from a Bayesian in their treatment of bad outcomes (test of $\gamma_B = 1$: p-value = 0.267). Consequently, we find that members tend to attribute good

[2]Results from our first-stage regression suggest that the appointment mechanisms are relevant instruments (F-statistic = 35.23).

outcomes more to luck and bad outcomes more to the DM's decision, and this effect is statistically significant (test of $\gamma_G = \gamma_B$: p-value = 0.042).

Table D4: IV regression of members' posterior beliefs

	Dependent variable: Logit(posterior)
	(1)
Variables	Pooled
δ : logit(interim belief)	0.792***
	(0.046)
γ_G : Good outcome $\times$ logit(p)	0.787***
	(0.056)
γ_B : Bad outcome $\times$ logit($1-p$)	0.929
	(0.064)
Observations	2,460
# subjects (clusters)	205
Test of $\gamma_G = \gamma_B$	
test statistic	−2.030
p-value	0.042

Robust standard errors clustered at the subject level in parentheses. This analysis excludes subjects classified as inconsistent or non-updaters.
*** p<0.01, ** p<0.05, * p<0.10. Null hypothesis is coefficient = 1.

D.3 Robustness checks of posterior beliefs analysis with inconsistent updaters and non-updaters

This section presents the analysis of members' updating behavior in Experiment 1 with the full sample (i.e., including both inconsistent updaters and non-updaters), as well as with the use of different criteria to exclude inconsistent updaters and non-updaters.

Figure D1 presents the distribution of subjects based on the number of inconsistent updates and non-updates throughout the experiment. A belief update is classified as *inconsistent* if the posterior belief is in the opposite direction to that predicted by Bayes' rule. A belief update is classified as a *non-update* if the posterior belief is equal to the interim belief. In the main analysis in the paper, we exclude a subject if 25% or more of their posterior beliefs are inconsistent or if they report a posterior belief equal to the interim belief across all six rounds of the experiment.

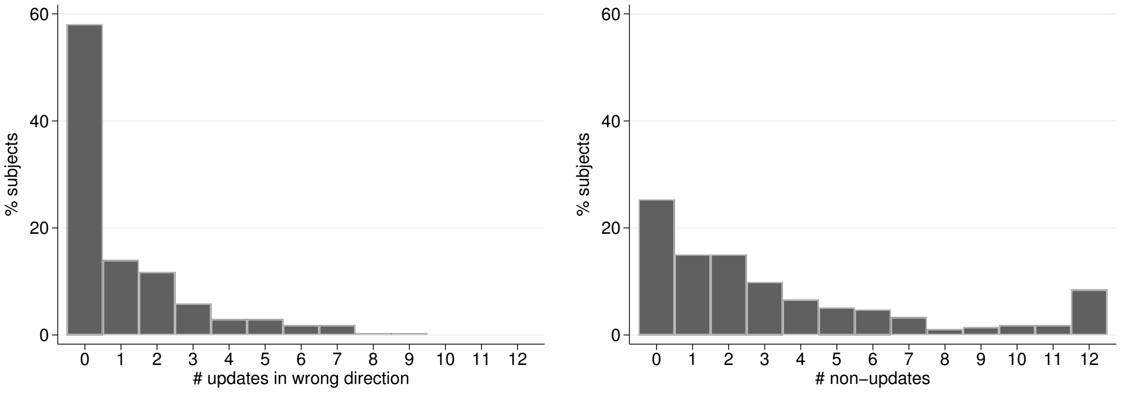

(a) Inconsistent updates (b) Non-updates

Figure D1: Distribution of inconsistent and non-updates by subjects

Table D5 presents results from the OLS estimation of equation (2) with the inclusion of these previously excluded inconsistent and non-updaters. Overall, we find that the inclusion of these subjects leads to an attenuation of the estimates for γ_G and γ_B. Consequently, at the pooled level (column 1), the estimates now reveal that members tend to attribute bad outcomes more to luck than a Bayesian would (test of $\gamma_B = 1$: p-value = 0.026). This bias is also present at the treatment level, although it is statistically significant in treatments RA, HA, and GA (p-values = 0.056, 0.087, and 0.011, respectively), but not in treatment LA (p-value = 0.379).

Table D5: Regression of members' posterior beliefs (entire sample)

	Dependent variable: Logit(posterior)				
	(1)	(2)	(3)	(4)	(5)
Variables	Pooled	RA	LA	HA	GA
δ : logit(interim belief)	0.701***	0.737***	0.709***	0.716***	0.539***
	(0.039)	(0.068)	(0.047)	(0.060)	(0.106)
γ_G : Good outcome × logit(p)	0.530***	0.548***	0.358***	0.618***	0.662***
	(0.064)	(0.086)	(0.094)	(0.083)	(0.093)
γ_B : Bad outcome × logit($1-p$)	0.848**	0.830*	0.903	0.867*	0.742**
	(0.068)	(0.089)	(0.110)	(0.078)	(0.100)
Observations	3,264	544	1,088	1,088	544
# subjects (clusters)	272	272	272	272	272
R-squared	0.550	0.620	0.606	0.488	0.382
Test of $\gamma_G = \gamma_B$					
test statistic	−3.218	−2.132	−3.376	−2.060	−0.550
p-value	0.001	0.034	0.001	0.040	0.583

Robust standard errors clustered at the subject level in parentheses. This analysis includes all subjects.
*** p<0.01, ** p<0.05, * p<0.10. Null hypothesis is coefficient = 1.

Despite the attenuation in the estimates for γ_G and γ_B, we still find statistically sig-

nificant evidence that members attribute good and bad outcomes asymmetrically. In particular, even with the inclusion of inconsistent updaters and non-updaters, the estimates in Table D5 suggest that members tend to attribute good outcomes more to luck and bad outcomes more to the DM's decision (i.e., $\gamma_G < \gamma_B$). This effect is statistically significantly at the pooled level (p-value = 0.001) and in treatments RA, LA, and HA (p-values = 0.034, 0.001, and 0.040, respectively).

We next consider different criteria for excluding non-updaters and inconsistent updaters. Table D6 presents estimates of members' updating behavior when we exclude rounds where a subject did not make an update for both a good and a bad outcome (column 1), and when we include only subjects who make an update in all six rounds of the task (column 2). Hence, in this table we vary our criteria for excluding subjects based on non-updates. Next, Table D7 presents estimates of members' updating behavior when we exclude subjects classified as non-updaters and at the same time consider different cut-offs as the criterion for excluding inconsistent updaters (columns 1-9). The maximum number of inconsistent updates by any subject is 9. Hence, column (10) only excludes subjects classified as non-updaters, i.e., those subjects who have not made a single update in all six rounds of the task. Note that column (3) is the criterion used in the paper and corresponds to column (1) of Table 4.

Both tables re-produce the results in Table 4 even when we consider different conditions for excluding non-updates or different thresholds for excluding inconsistent updaters or non-updaters. Importantly, base-rate neglect is always observed (in the first row of both tables). Moreover, in both tables, we systematically observe that good outcomes are attributed to luck (second row), and that good and bad outcomes are treated asymmetrically (last row). Hence, our results are robust to using different criteria for excluding both inconsistent and non-updaters.

Table D6: Regression of members' posterior beliefs (different criteria for excluding non-updaters)

	Dependent variable: Logit(posterior)	
	(1)	(2)
Variables	Include only subject-rounds with updates	Include only subjects with updates in all rounds
δ : logit(interim belief)	0.465***	0.546***
	(0.047)	(0.136)
γ_G : Good outcome $\times$ logit(p)	0.715***	0.674**
	(0.078)	(0.129)
γ_B : Bad outcome $\times$ logit($1-p$)	0.957	1.148
	(0.078)	(0.097)
Observations	2,692	828
# subjects (clusters)	249	69
R-squared	0.346	0.465
Test of $\gamma_G = \gamma_B$	-0.242	-0.474
test statistic	-2.010	-2.558
p-value	0.046	0.013

Robust standard errors clustered at the subject level in parentheses. This analysis considers different cut-offs for non-updates. Column (1) includes only subject-rounds with belief updates. Column (2) includes only subjects who have revised their beliefs in all rounds of the investment tasks.

*** p<0.01, ** p<0.05, * p<0.10. Null hypothesis is coefficient = 1.

Table D7: Regression of members' posterior beliefs (different criteria for excluding inconsistent updaters)

	Dependent variable: Logit(posterior)									
	Criteria for excluding subjects: # inconsistent updates									
	(1)	(2)	(3)	(4)	(5)	(6)	(7)	(8)	(9)	(10)
Variables	≥ 1	≥ 2	≥ 3	≥ 4	≥ 5	≥ 6	≥ 7	≥ 8	≥ 9	≥ 10
δ : logit(interim belief)	0.783***	0.759***	0.695***	0.667***	0.650***	0.652***	0.653***	0.656***	0.656***	0.656***
	(0.040)	(0.038)	(0.039)	(0.039)	(0.042)	(0.041)	(0.041)	(0.041)	(0.041)	(0.041)
γ_G : Good outcome $\times$ logit(p)	0.786***	0.728***	0.751***	0.752***	0.742***	0.661***	0.653***	0.607***	0.606***	0.602***
	(0.060)	(0.055)	(0.051)	(0.052)	(0.052)	(0.064)	(0.066)	(0.069)	(0.069)	(0.069)
γ_B : Bad outcome $\times$ logit($1-p$)	1.072	1.030	0.966	0.902	0.873*	0.923	0.913	0.916	0.909	0.905
	(0.088)	(0.074)	(0.067)	(0.068)	(0.067)	(0.072)	(0.072)	(0.072)	(0.072)	(0.072)
Observations	1,620	2,076	2,460	2,652	2,748	2,844	2,904	2,964	2,976	2,988
# subjects (clusters)	135	173	205	221	229	237	242	247	248	249
R-squared	0.710	0.692	0.608	0.565	0.548	0.524	0.518	0.504	0.503	0.503
Test of $\gamma_G = \gamma_B$										
t-statistic	−3.401	−4.216	−3.190	−2.058	−1.771	−2.575	−2.499	−2.862	−2.815	−2.825
p-value	0.001***	< 0.001***	0.002***	0.041**	0.078*	0.011**	0.013**	0.005***	0.005***	0.005***

Robust standard errors clustered at the subject level in parentheses. This analysis excludes subjects classified as non-updaters (using the strictest criteria) and considers different cut-offs for inconsistent updaters. Column (3) is the benchmark used in the paper. The maximum number of inconsistent updates by any subject is 9. Hence, column (10) only excludes non-updaters.

*** p<0.01, ** p<0.05, * p<0.10. Null hypothesis is coefficient = 1.

D.4 Heterogeneity in updating behavior

Estimates at the pooled level may mask heterogeneity in members' updating behavior. To explore this further, Table D8 presents the results from both a 2-component (column 1) and 3-component (column 2) finite mixture model analysis of members' updating behavior at the pooled level.[3]

In both models considered in Table D8, component 1 constitute the majority of updates in the sample (88.9% of the updates in the 2-component and 65.9% of the updates in the 3-component model). This component is characterized by a low level of base-rate neglect and under-responsiveness to both good and bad outcomes. Within this group of belief updates, in relative terms, members attribute good outcomes more to luck and bad outcomes more to the DM's decision, although this difference is statistically significant in the 2-component model (p-value = 0.001) but not in the 3-component model (p-value = 0.190).

The estimates in the table reveal that belief updates in the remaining sample suffer from a higher level of base-rate neglect. Moreover, the under-responsiveness to outcomes is no longer present within this group of updates. Instead, group members are over-responsive to outcomes in component 2 (11.1% of the updates in the 2-component and 4.8% of the updates in the 3-component model). In addition, a third sub-group is identified in the 3-component model (constituting 29.4% of the sample) where members respond to outcomes like a Bayesian.

Overall, our finite mixture model analysis suggests that there is heterogeneity in members' updating behavior. Although members consistently suffer from base-rate neglect, for most updates this is at a modest level. Moreover, the majority of belief updates in the sample is characterized by under-responsiveness to the DM's outcomes and an asymmetric attribution of the DM's outcomes to his/her decision and luck.

[3]We also consider a 4-component model which does not change our main conclusions and does not provide further insight.

Table D8: Finite mixture model for updating behavior

	Dependent variable: Logit(posterior)	
	2-Component Model	3-Component Model
	(1)	(2)
**Component 1**		
δ : logit(interim belief)	0.936***	0.972***
	(0.011)	(0.005)
γ_G : Good outcome $\times$ logit(p)	0.535***	0.431***
	(0.031)	(0.031)
γ_B : Bad outcome $\times$ logit($1-p$)	0.668***	0.477***
	(0.044)	(0.040)
Test of $\gamma_G = \gamma_B$		
t-statistic	−3.47	−1.31
p-value	0.001	0.190
**Component 2**		
δ : logit(interim belief)	0.148***	−0.109***
	(0.086)	(0.137)
γ_G : Good outcome $\times$ logit(p)	1.936**	3.566***
	(0.405)	(0.807)
γ_B : Bad outcome $\times$ logit($1-p$)	1.945**	2.942***
	(0.407)	(0.642)
Test of $\gamma_G = \gamma_B$		
t-statistic	−0.02	0.70
p-value	0.984	0.485
**Component 3**		
δ : logit(interim belief)		0.302***
		(0.031)
γ_G : Good outcome $\times$ logit(p)		1.029
		(0.054)
γ_B : Bad outcome $\times$ logit($1-p$)		1.103
		(0.067)
Test of $\gamma_G = \gamma_B$		
t-statistic		−0.89
p-value		0.372
**Latent Class Marginal Probabilities**		
μ_1	0.889	0.659
	(0.020)	(0.028)
μ_2	0.111	0.048
	(0.020)	(0.009)
μ_3		0.294
		(0.027)
**Model Fit**		
Log likelihood	−3317.86	−3028.11
AIC	6653.720	6084.223
BIC	6705.991	6165.534

Robust standard errors clustered at the subject level in parentheses. This analysis excludes subjects classified as inconsistent or non-updaters.
*** p<0.01, ** p<0.05, * p<0.10. Null hypothesis is coefficient = 1.

D.5 Subjects' behavior in Experiment 2

Table D9 presents summary statistics of subjects' characteristics in Experiment 1 and Experiment 2. The table reveals significant differences in the subject pool between Experiment 1 and Experiment 2, but not between treatments S and D in Experiment 2 (p-values for test of joint equality are < 0.001 and 0.750, respectively). On average, subjects in Experiment 2 are older (p-value < 0.001), less likely to be Australian (p-value < 0.001) or majoring in economics (p-value $= 0.001$), more likely to be a postgraduate student (p-value < 0.001), and more experienced with economics experiments (p-value < 0.001). However, the subjects do not differ on their decisions in the dictator game or in the risk task between Experiment 1 and Experiment 2 (p-values $= 0.125$ and 0.565, respectively).

Table D9: Subjects' characteristics in Experiment 1 and Experiment 2

	Exp 2				
	Treatment S	Treatment D	Exp 1	S vs. D	Exp 1 vs. Exp 2
	(1)	(2)	(3)	p-value	p-value
Age	24.963	25.388	21.478	0.379	< 0.001***
	[4.735]	[6.092]	[5.843]		
Female	0.596	0.592	0.537	0.936	0.135
	[0.512]	[0.502]	[0.521]		
Economics major	0.064	0.078	0.140	0.554	0.001***
	[0.245]	[0.268]	[0.347]		
Postgraduate student	0.572	0.515	0.246	0.201	< 0.001***
	[0.496]	[0.501]	[0.432]		
Australian	0.226	0.248	0.971	0.568	< 0.001***
	[0.419]	[0.433]	[0.169]		
# past experiments	2.754	2.874	0.908	0.646	< 0.001***
	[2.380]	[3.458]	[1.757]		
Amount transferred in DG	29.906	30.785	32.809	0.667	0.125
as % of endowment	[21.739]	[23.569]	[21.117]		
Amount invested in RT	71.104	70.680	72.103	0.860	0.565
as % of endowment	[27.175]	[25.414]	[28.220]		
Observations	297	206	272		

Standard deviations in parentheses.
DG: Dictator Game; RT: Risk Task.
*** p<0.01, ** p<0.05, * p<0.10.

We next examine whether members' hypothetical effort choices as DMs in treatment S are consistent with their incentivized decisions in the dictator game. Table D10 presents marginal-effect estimates from probit regressions of members' hypothetical choices as DMs in round 1 only (column 1) and in all rounds of the task (column 2). We find a statistically significant and positive relationship between members' hypothetical effort

choices as DMs and their incentivized decisions in the dictator game (p-values < 0.001 in both columns). A member who transfers 1% more of their endowment to their matched partner in the dictator game is 0.7% more likely to state that they would have chosen high effort as a DM in round 1 of the task. Across all rounds of the investment task, these members are on average 0.5% more likely to state that they would have chosen high effort as DMs.

Table D10: Regression of members' hypothetical effort choices as DMs in treatment S

	Dependent variable: =1 if member would have chosen e_H as DM	
	Round 1 only	All rounds
Variables	(1)	(2)
% endowment transferred in DG	0.007***	0.005***
	(0.001)	(0.001)
% endowment invested in RT	0.001	0.000
	(0.001)	(0.001)
Game 2	0.043	0.034
	(0.072)	(0.030)
Game 0	0.069	0.010
	(0.072)	(0.034)
Order Effects	N	Y
Observations	198	594
# subjects (clusters)	198	198

Marginal effects of probit model reported. Robust standard errors in parentheses. Standard errors are clustered at the subject level. Game 1 is the reference group in both regressions. DG: Dictator Game; RT: Risk Task.
*** p<0.01, ** p<0.05, * p<0.10.

We next compare members' updating behavior between treatments S and D, and between Experiment 1 and Experiment 2. Table D11 presents parameter estimates of equation (2) by members' effort choices in treatments S and D, respectively. In the table, we present p-values of pairwise comparisons of the estimates both within each treatment and between treatments. A pairwise comparison of the estimates in columns (3) and (4) reveal that, when subjects play both roles in the experiment, those who choose low effort as DMs are more likely to, as members, attribute good outcomes to luck than those who choose high effort as DMs (p-value = 0.024). This is consistent with members' behavior both in treatment S and in Experiment 1. Moreover, when we examine the updating behavior of subjects separately based on their effort choices as DMs, we do not find any

systematic differences between treatments S and D in their attribution of both good and bad outcomes (those who choose low effort: p-values = 0.656 and 0.459, respectively, and those who choose high effort: p-values = 0.264 and 0.190, respectively).[4]

Table D12 presents parameter estimates of members' updating behavior at the pooled level in treatments S and D (columns 1 and 2). Pairwise comparisons of the estimates reveal that there are no systematic differences in members' updating behavior between the two treatments. Hence, in column (3), we report the parameter estimates by pooling together the subjects in both treatments. The estimates reveal that, overall in Experiment 2, members tend to attribute good outcomes more to luck as compared to a Bayesian (p-value = 0.086). When we compare the members' updating behavior in Experiment 2 with those in Experiment 1 (column 1 of Table 4), we do not find any statistically significant differences in the members' attribution of both good and bad outcomes (p-values = 0.493 and 0.475, respectively), although we find that members in Experiment 2 suffer from stronger base-rate neglect than those in Experiment 1 (p-value = 0.008).

[4]Note that subjects who choose high effort as DMs in treatment D suffer from a stronger base-rate neglect than those who would have chosen high effort hypothetically as DMs in treatment S (p-value = 0.054).

Table D11: Regression of members' posterior beliefs based on effort choice as DMs in Experiment 2 (treatments S and D)

	Dependent variable: Logit(posterior)							
	Treatment S			Treatment D			S vs. D	
	(1)	(2)	(1) vs. (2)	(3)	(4)	(3) vs. (4)	(1) vs. (3)	(2) vs. (4)
Variables	Low effort	High effort	p-value	Low effort	High effort	p-value	p-value	p-value
δ : logit(prior belief)	0.483***	0.607***	0.254	0.571***	0.339***	0.098*	0.440	0.054*
	(0.079)	(0.077)		(0.083)	(0.115)			
γ_G : Good outcome $\times$ logit(p)	0.601*	1.068	0.085*	0.730	1.383*	0.024**	0.656	0.264
	(0.210)	(0.173)		(0.201)	(0.223)			
γ_B : Bad outcome $\times$ logit($1-p$)	1.181	0.759	0.082*	0.989	1.074	0.734	0.459	0.190
	(0.188)	(0.171)		(0.179)	(0.169)			
Observations	656	232		694	320			
# subjects (clusters)	137	71		147	90			
R-squared	0.392	0.488		0.432	0.323			

Robust standard errors clustered at the subject level in parentheses. This analysis excludes subjects classified as inconsistent or non-updaters.
Columns (1) and (2) control for members' hypothetical effort choices in treatment S, and are the same as columns (3) and (4) of Table 6.
*** p<0.01, ** p<0.05, * p<0.10. Null hypothesis in columns (1)-(4) is coefficient = 1.

Table D12: Regression of members' posterior beliefs at pooled level in Experiment 2 (treatments S and D)

	Dependent variable: Logit(posterior)				
	Treatment S	Treatment D	S + D	(1) vs. (2)	(3) vs. Exp 1[a]
Variables	(1)	(2)	(3)	p-value	p-value
δ : logit(prior belief)	0.525***	0.546***	0.535***	0.815	0.008***
	(0.063)	(0.064)	(0.045)		
γ_G : Good outcome $\times$ logit(p)	0.803	0.845	0.827*	0.836	0.493
	(0.146)	(0.139)	(0.100)		
γ_B : Bad outcome $\times$ logit($1-p$)	0.990	1.107	1.052	0.537	0.475
	(0.145)	(0.122)	(0.094)		
Observations	888	1,014	1,902		
# subjects (clusters)	148	169	317		
R-squared	0.399	0.400	0.399		

Robust standard errors clustered at the subject level in parentheses. This analysis excludes subjects classified as inconsistent or non-updaters.

(a) This refers to the estimates reported in column (1) of Table 4.

*** p<0.01, ** p<0.05, * p<0.10. Null hypothesis in columns (1)-(3) is coefficient = 1.

E Detailed explanation of estimation strategy for posterior beliefs

In this section, we explain in detail the interpretations of the parameters presented in Section 2.3.3.

We first express posterior beliefs in terms of log likelihood ratios. We have

$$\log\left(\frac{\phi_i^{\Psi}|_{Q_H}}{1-\phi_i^{\Psi}|_{Q_H}}\right) = \log\left(\frac{\mu_i^{\Psi}}{1-\mu_i^{\Psi}}\right) + \log\left(\frac{p}{1-p}\right), \tag{E1}$$

and

$$\log\left(\frac{\phi_i^{\Psi}|_{Q_L}}{1-\phi_i^{\Psi}|_{Q_L}}\right) = \log\left(\frac{\mu_i^{\Psi}}{1-\mu_i^{\Psi}}\right) + \log\left(\frac{1-p}{p}\right). \tag{E2}$$

By letting $\text{logit}(x) \equiv \log\left(\frac{x}{1-x}\right)$, we can jointly express (E1) and (E2) as

$$\text{logit}(\phi_i^{\Psi}|_Q) = \text{logit}(\mu_i^{\Psi}) + I(Q = Q_H)\cdot \text{logit}(p) + I(Q = Q_L)\cdot \text{logit}(1-p), \tag{E3}$$

where $I(\cdot)$ is an indicator function.

Equation (2) in the paper is obtained by augmenting equation (E3) in the following way:

$$\text{logit}(\hat{\phi_i^{\Psi}}|_Q) = \delta\, \text{logit}(\hat{\mu_i^{\Psi}}) + \gamma_G\, I(Q = Q_H)\cdot \text{logit}(p) + \gamma_B\, I(Q = Q_L)\cdot \text{logit}(1-p) + \varepsilon_i, \tag{E4}$$

where ε_i captures non-systematic errors. This specification allows us to determine the weights members place on their interim beliefs and the signals they receive. Note that $\delta = \gamma_G = \gamma_B = 1$ equates (E4) to (E3). This is the case where there is no bias in belief updating.

Any deviation in the parameters from 1 is interpreted as non-Bayesian updating behavior. Specifically, δ captures the weight that a group member places on his/her interim belief in the updating process, γ_G captures the extent to which a member responds to a signal of good outcome from the DM, and γ_B captures the extent to which a member responds to a signal of bad outcome from the DM. We use Figures E1 and E2 to explain these parameters in more detail.

Figure E1 shows the implications of different values of δ on the relationship between the member's posterior and interim beliefs, conditional on observing a good outcome and holding γ_G constant (at 1).[5] Note that δ corresponds to the slope of the linear regression. If $\delta < 1$, then the member suffers from *base-rate neglect* in that s/he places too little weight on his/her interim belief. To see this, consider a member whose interim belief μ_A is less than 0.5. This corresponds to $\text{logit}(\mu_A) < 0$ in Figure E1. Hence, the member believes that the DM is more likely to have chosen low effort. When Q_H is observed, the signal contradicts with the interim belief. However, s/he arrives at a posterior belief that

[5]A similar analysis can be done for the case where a bad outcome is observed.

is greater than that of a Bayesian (i.e., point A' instead of point A). In other words, the member neglects his/her interim belief and over-updates in response to receiving a signal that contradicts with what s/he initially believes to be true.[6]

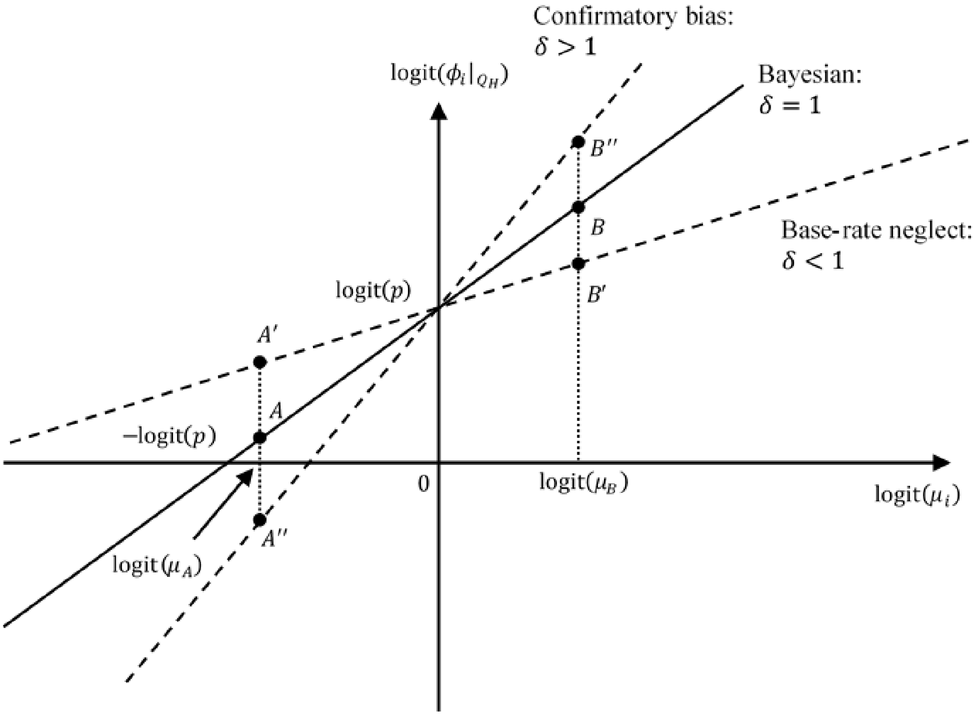

Figure E1: Interpretation of δ given Q_H observed and $\gamma_G = 1$

Conversely, $\delta > 1$ implies that the member suffers from *confirmatory bias* in that s/he places too much weight on his/her interim belief. To see this, consider a member whose interim belief μ_B is greater than 0.5, i.e., $\text{logit}(\mu_B) > 0$ in Figure E1. When Q_H is observed, the signal confirms the interim belief. However, his/her posterior belief is at point B'' instead of point B. Hence, the member over-updates relative to a Bayesian when s/he receives a signal that confirms what s/he initially believes to be true.[7]

Figure E2 shows the implications of different values of γ_G on the relationship between the member's posterior and interim beliefs.[8] Note that γ_G and γ_B correspond to the intercepts of the regression conditional on the signal received by the member. If $\gamma_G > 1$, then the member is, on average, over-responsive to good signals relative to a Bayesian, and tends to arrive at a posterior that is higher than that of a Bayesian. Specifically,

[6]Now consider a member whose interim belief μ_B is greater than 0.5. After observing Q_H, a signal that confirms this belief, suppose that his/her posterior belief is at B'. This implies that a member who suffers from base-rate neglect does not update as much as a Bayesian would when s/he receives a signal that confirms his/her interim belief.

[7]Alternatively, consider a member whose interim belief μ_A is less than 0.5. After observing Q_H, a signal that contradicts with this belief, suppose that his/her posterior belief is at A''. This implies that a member who suffers from confirmatory bias does not update as much as a Bayesian would when s/he receives information that contradicts with his/her interim belief.

[8]A similar analysis can be done for the case where a bad outcome is observed.

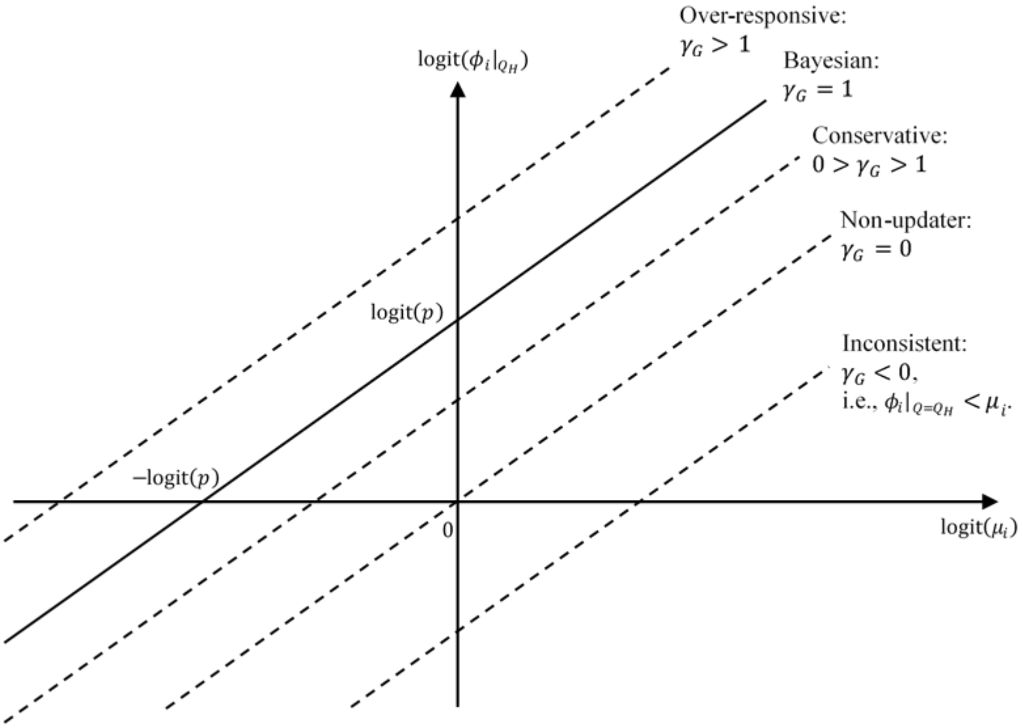

Figure E2: Interpretation of γ_G given Q_H observed and $\delta = 1$

the biased member attributes good outcomes more to the DM's decision as compared to an unbiased Bayesian member. On the other hand, if $\gamma_G < 1$, then the member is conservative in his/her response to good signals, and tends to arrive at a posterior that is lower than that of a Bayesian on average. In this case, the biased member attributes good outcomes more to luck as compared to an unbiased Bayesian member. Figure E2 also shows what happens when $\gamma_G = 0$ or $\gamma_G < 0$, which correspond to a non-updater and an inconsistent updater, respectively.

Finally, we can also capture asymmetric updating of beliefs, i.e., asymmetric attribution of outcomes to the DM's decision (effort choice) and luck. If $\gamma_G > \gamma_B$, then the member is more likely to attribute a good outcome to the DM's decision and a bad outcome to luck. Conversely, if $\gamma_G < \gamma_B$, then the member is more likely to attribute a bad outcome to the DM's decision and a good outcome to luck.
